# Effect of aging on the formation and growth of colonic epithelial organoids by changes in cell cycle arrest through TGF-β-Smad3 signaling

**DOI:** 10.1186/s41232-023-00282-6

**Published:** 2023-07-13

**Authors:** Min Kyoung Jo, Chang Mo Moon, Hyeon-Jeong Jeon, Yerim Han, Eun Sook Lee, Ji-Hee Kwon, Kyung-Min Yang, Young-Ho Ahn, Seong-Eun Kim, Sung-Ae Jung, Tae Il Kim

**Affiliations:** 1 Department of Internal Medicine, College of Medicine, Ewha Womans University, 1071 Anyangcheon-ro, Yangcheon-gu, Seoul 07985 Republic of Korea; 2 Inflammation-Cancer Microenvironment Research Center, College of Medicine, Ewha Womans University, 25, Magokdong-ro 2-gil, Gangseo-gu, Seoul 07804 Republic of Korea; 3 Division of Gastroenterology and Department of Internal Medicine, Yonsei University College of Medicine, 50-1 Yonsei-Ro, Seodaemun-Gu, Seoul, 03722 Republic of Korea; 4 Medpacto Inc., Seoul, Republic of Korea; 5 Department of Molecular Medicine, College of Medicine, Ewha Womans University, Seoul, Republic of Korea

**Keywords:** Aging, Organoid, TGF-β, Smad3

## Abstract

**Background:**

This study aimed to investigate how aging alters the homeostasis of the colonic intestinal epithelium and regeneration after tissue injury using organoid models and to identify its underlying molecular mechanism.

**Methods:**

To investigate aging-related changes in the colonic intestinal epithelium, we conducted organoid cultures from old (older than 80 weeks) and young (6–10 weeks) mice and compared the number and size of organoids at day 5 of passage 0 and the growth rate of organoids between the two groups.

**Results:**

The number and size of organoids from old mice was significantly lower than that from young mice (*p* < 0.0001) at day 5 of passage 0. The growth rate of old-mouse organoids from day 4 to 5 of passage 0 was significantly slower than that of young-mouse organoids (2.21 times vs. 1.16 times, *p* < 0.001). RNA sequencing showed that TGF-β- and cell cycle-associated genes were associated with the aging effect. With regard to mRNA and protein levels, Smad3 and p-Smad3 in the old-mouse organoids were markedly increased compared with those in the young-mouse organoids. Decreased expression of ID1, increased expression of p16^INK4a^, and increased cell cycle arrest were observed in the old mouse-organoids. Treatment with SB431542, a type I TGF-β receptor inhibitor, significantly increased the formation and growth of old-mouse organoids, and TGF-β1 treatment markedly suppressed the formation of young-mouse organoids. In the acute dextran sulfate sodium-colitis model and its organoid experiments, the colonic epithelial regeneration after tissue injury in old mice was significantly decreased compared with young mice.

**Conclusions:**

Aging reduced the formation ability and growth rate of colonic epithelial organoids by increasing cell cycle arrest through TGF-β-Smad3-p16^INK4a^ signaling.

**Supplementary Information:**

The online version contains supplementary material available at 10.1186/s41232-023-00282-6.

## Background

Aging is defined as a time-dependent process of decreasing physiological function and accumulation of damage in tissues or cells due to multiple factors over a human lifetime [1, 2]. The loss of cellular homeostasis and alterations in various intracellular molecules are related to the aging process at the cellular level [3]. Furthermore, aging attenuates tissue functions and is closely associated with the etiology of endocrine diseases, cardiovascular disease, immunologic diseases, and cancer [4].

The intestine is one of the most critical epithelia in the body, and its epithelium plays an important role in many physiological functions, including nutrient digestion, absorption, and secretion [5–7]. Furthermore, it acts as a chemical barrier to the gut microbiota and modulates immune responses [5–7]. Aging, in which the physiological activity of the human intestinal epithelium slowly decreases, is associated with decreased barrier function [8], malabsorption [9], overgrowth of small intestinal bacteria [10], and an increased risk of gastrointestinal cancer [11, 12].

Most studies about aging are motivated by the need to investigate the human aging process, but their methodology has many limitations. Although it is necessary to observe the changes related to aging in cells, tissues, and organs over time, most aging experimental models have balanced issues between biological relevance and practicality [4]. From this point of view, organoid culture methods, which facilitate the development of mini-organ models in vitro, have the potential to overcome many of the limitations of traditional aging models [4]. Since organoids have the advantage of being used to experiment with aging tissues and cells, they are a suitable model for observing aging phenotypes [4]. However, few studies have investigated the effect of aging on the colonic intestinal epithelium and its underlying mechanism using an organoid model.

Therefore, in this study, we aimed to investigate whether aging alters the intestinal epithelial homeostasis and regeneration after tissue injury and to evaluate what intestinal phenotype related to aging is present in the organoid culture system. We subsequently sought to identify the molecular mechanism underlying the effect of aging on the changes in intestinal epithelial function.

## Methods

### Mice

This study was approved and conducted in accordance with the regulations and guidelines of the Institutional Animal Care and Use Committee of Ewha Womans University College of Medicine. Young and old male C57BL/6 mice were purchased from Orient Bio (Seongnam, Korea) and maintained in the animal facility of Ewha Womans University. 6- to 10-week-old mice were used as young mice, and mice older than 20 months old were used as old mice.

### Isolation of murine colon crypts

Murine intestinal crypts were harvested from young and old mice. Harvesting and isolation of crypts were performed with minor modifications according to a previously described protocol [13]. Murine colon crypts were rinsed with cold Dulbecco's phosphate-buffered saline (PBS; Capricorn Scientific, Ebsdorfergrund, Germany) and incubated in 0.7% sodium hypochlorite solution (NaOCl; Sigma Aldrich, Saint Louis, USA) in PBS for 5 min and then incubated in 3 mM ethylenediaminetetraacetic acid (EDTA; Sigma Aldrich) and 0.5 mM 1,4-dithiothreitol (DTT; Roche, Mannheim, Germany) in PBS for 40 min. After removal of the EDTA buffer, crypts were then detached from the basal membrane by vigorous shaking in cold PBS. Next, all crypts were filtered with a 70-µm cell strainer (BD Biosciences, Bedford, MA, USA) to yield a crypt pellet that was resuspended in 10 ml of PBS and centrifuged twice. Isolated crypts were counted at 500 crypts per 20 μl and embedded in a collagen matrix that consisted of cell matrix type I-A porcine tendon collagen (Component A; Nitta Gelatin Inc., Osaka, Japan), 10 × Ham's F12 (Component B), and a buffer solution of 2.2 g of NaHCO_3_ in 100 mL of 0.05 N NaOH and 200 mM HEPES (Component C) in an 8:1:1 (A:B:C) volume. Approximately 20 μl of droplets of collagen gel with crypts were plated on flat-bottom 48-well plates (Corning Incorporated, NY, USA), and 50 μl of droplets of collagen gel with crypts were plated onto a flat-bottom 24-well plate (Corning Incorporated) and allowed to solidify for 30 to 40 min in a 37 ℃ incubator and cultured in a modified form of the medium as described previously for 5–7 days in a humid 37 ℃ incubator. The medium was changed every two days. Organoids were quantified on days 4 and 5 of culture, unless otherwise specified.

### Colonic organoid culture

Colonic crypts were isolated from mice and cultured as described previously [13]. Briefly, a total of 500 crypts were mixed with 20 μl of collagen and plated in 48-well plates. After the collagen polymerized, 500 μl of the organoid culture medium was added to each well. For the production of the organoid culture medium, L-WRN cells were cultured in DMEM high glucose (Thermo Fisher, Waltham, MA, USA) supplemented with 10% fetal bovine serum (FBS; Capricorn Scientific GmbH), 1 × penicillin–streptomycin (WelGENE Inc., Daegu, Korea), 500 μg/ml gentamicin (Gibco, Grand Island, NY, USA), and 500 μg/ml hygromycin B (Thermo Fisher Scientific). The L-WRN (CRL-3276) cell line was obtained from the American Type Culture Collection (ATCC). The 50% L-WRN conditioned media (CM) that replaced R-spondin, Wnt3A, and Noggin was made as described [13]. L-WRN was next cultured with Advanced DMEM/F12 (Gibco) supplemented with 20% FBS, 1 × penicillin–streptomycin, and 20 μM L-Glutamine (Gibco) to make L-WRN CM. Finally, organoid media was contained with 50% L-WRN CM and 50% basal media (1:1) composed of 1 × penicillin–streptomycin, 50 μg/mL gentamicin (Life Technologies), 1 × N2 (Life Technologies), B27 (without retinoic acid, Life Technologies), and 1 mM N-Acetyl-L-cysteine (Sigma-Aldrich) with added 20 ng/mL mouse EGF (ProSpec, Rehovot, Israel), 50 ng/mL mouse HGF (ProSpec), 100 μg/mL primocin (Invivogen, San Diego, CA, USA), and 3 μM CHIR 99021 (ProSpec).

### Colonic organoid culture from DSS-colitis mice

Dextran sulfate sodium (DSS)-colitis mice were sacrificed on day 16. Colonic crypts were isolated from mice and cultured as described previously [13]. Briefly, a total of 2000 crypts were mixed with 20 μl of collagen and plated in 48-well plates. For the RNA extraction, 50 μl of droplets of collagen gel with crypts were plated onto a flat-bottom 24-well plate. After the collagen polymerized, 200–500 μl of the organoid culture medium was added to each well. The medium was changed every 2 days. Organoids from DSS-colitis mice were quantified on days 6 and 7 of culture.

### Statistical analyses

Data are expressed as the mean ± standard error of the mean (SEM) or mean ± standard deviation (SD). All analyses were carried out using Graph-Pad Prism 8.0 software (Graph Pad Software, a Jolla, CA, USA) and SPSS software (version 22.0, Chicago, IL, USA). Data are expressed as mean ± SEM. or ± SD, and p-values < 0.05 were considered significant. The Mann–Whitney t-test for nonparametric test for parametric data was used to determine statistical significance in this study. (^*^, *p* < 0.05; ^**^, *p* < 0.01; ^***^, *p* < 0.001; ^****^ *p* < 0.0001).

Other detailed methods for experiments (colonic organoid culture in Matrigel, quantification of the number and growth rate of organoids, passage of colonic epithelial organoids, RNA isolation and quantitative real-time PCR, Western blot, cell cycle analysis, flow cytometry, immunohistochemistry, RNA sequencing library and bioinformatic analysis, DSS colitis mouse experiment, and construction of a protein–protein interaction (PPI) network are described in the online Supplementary materials and methods.

## Results

### Impact of aging on the formation and growth of colonic epithelial organoids

**Fig. 1:**
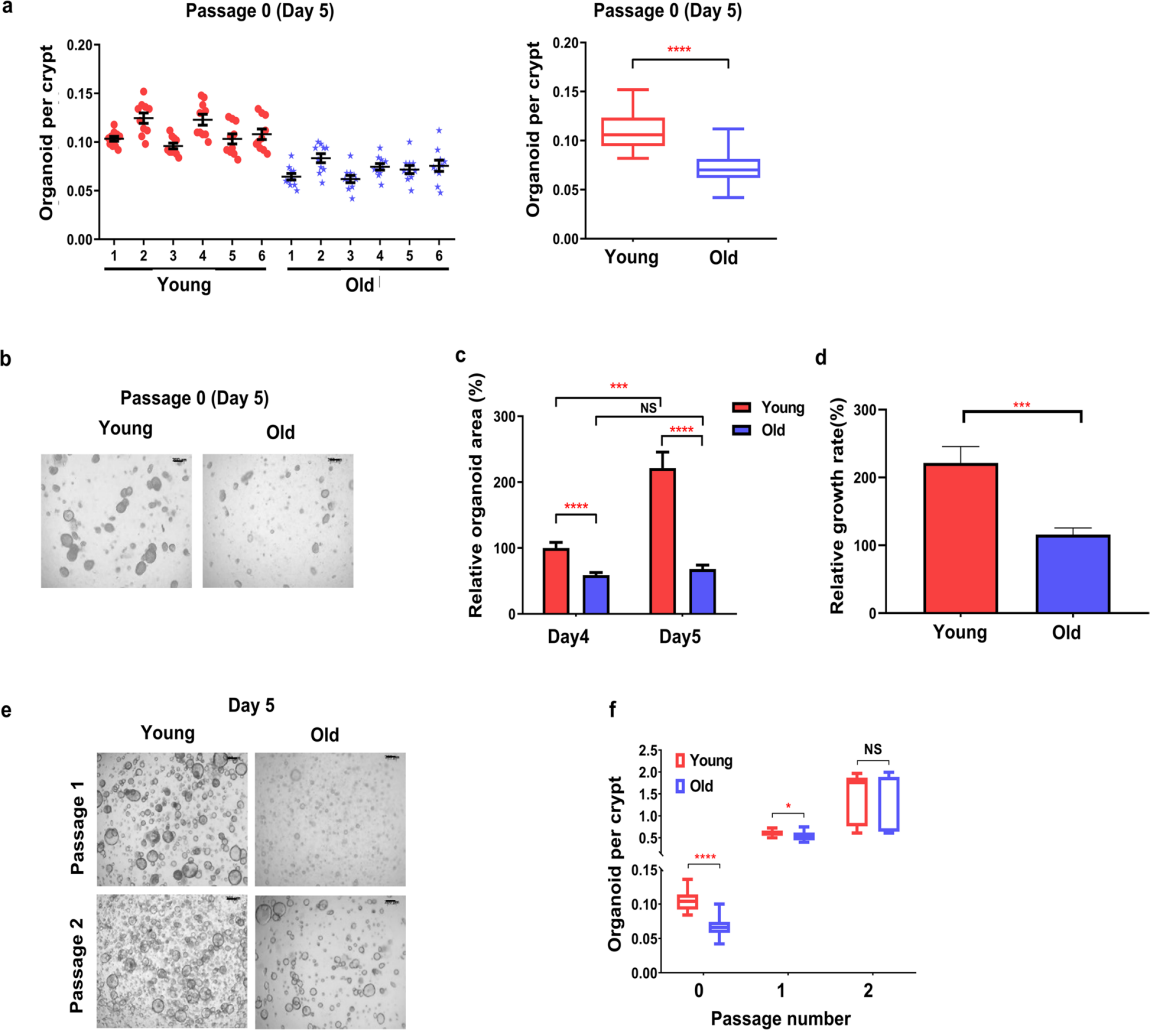
Effects of aging on the formation and growth of the colonic epithelial organoids. **a** Number of organoids derived from young and old mice that were cultured at a density of 500 crypts per well in the primary organoid culture (left) and the average number in each group (right). Data are reported as the mean ± standard deviation (SD) of 30 wells (5 wells per mouse; 6 mice per group). **b** Representative images of young- and old-mouse organoids on day 5 of passage 0 after seeding. Scale bar represents 200 μm. **c** Changes in the areas of young- and old-mouse organoids at days 4 and 5. The represented percentage is relative to young-mouse organoids on day 4. Data are reported as the mean ± SD of 30 organoids (5 organoids per mouse; 6 mice per group). **d** The growth rate of young- and old-mouse organoids (5 organoids per mouse; 6 mice per group). Data are reported as the mean ± SD. **e** Representative images of young- and old-mouse organoids on day 5 of passages 1 and 2 (Scale bar = 200 μm). **f** Organoid formation of 500 crypts per well in the primary organoids on day 5 of passages 0, 1 and 2 (3 mice per group). Significance was calculated by the Mann–Whitney t-test. ^*^p < 0.05, ^**^p < 0.01, ^***^p < 0.001, ^****^p < 0.0001

To assess the effect of age on intestinal homeostasis, we divided the mice into two groups according to age (old mice were older than 20 months old, and young mice were 6 − 10 weeks age) and compared them using a colonic epithelial organoid model. We assessed the initial formation and growth of colonic organoids at passage 0 between young and old mice. When we cultured 500 crypts per group in each experiment, the number of organoids (per 500 crypts) from old mice (0.072 ± 0.015) was significantly lower than that from young mice (0.110 ± 0.018, *p* < 0.0001) (Fig. 1a).

The organoid size between the two groups was also different (Fig. 1b, c). To compare the organoid size between the two groups, we measured the organoid area from 5 organoids per mouse (for 6 mice per group) on day 4 and 5 of passage 0 using Image J. We set the organoid area obtained from young mice on day 4 of passage 0 to 100%. The relative organoid area from old mice (58.9 ± 22.2%) was significantly smaller than that from young mice (100.0 ± 47.6%) on day 4 (*p* < 0.0001). The organoid size in both groups showed the same pattern on day 5 (221.3 ± 133.8% in young mice vs. 68.2 ± 32.0% in old mice, *p* < 0.0001) (Fig. 1c). To compare the organoid growth rate, the change in the organoid area from days 4 to 5 was compared between young- and old-mouse organoids. As a result, young-mouse organoids on day 5 grew 2.21 times compared to those at day 4, but old-mouse organoids grew by 1.16 times. The organoid area of young mice on day 5 was significantly larger than that on day 4 (*p* < 0.001), while there was no difference between the organoid area of old mice on day 4 and 5 (*p* = 0.254) (Fig. 1c). The growth rate of colonic organoids from day 4 to 5 in young mice was significantly faster than that in old mice (*p* < 0.001) (Fig. 1d). However, the difference in the organoid formation disappeared when organoid cultures were passaged for maintenance. There was no difference in the number of organoids between young and old mice at passage 2 (Fig. 1e, f). Taken together, these results suggest that aging significantly reduces the forming ability and growth rate of colonic epithelial organoids.

### TGF-β-Smad3 signaling is associated with the effect of aging on colonic epithelial organoids

**Fig. 2:**
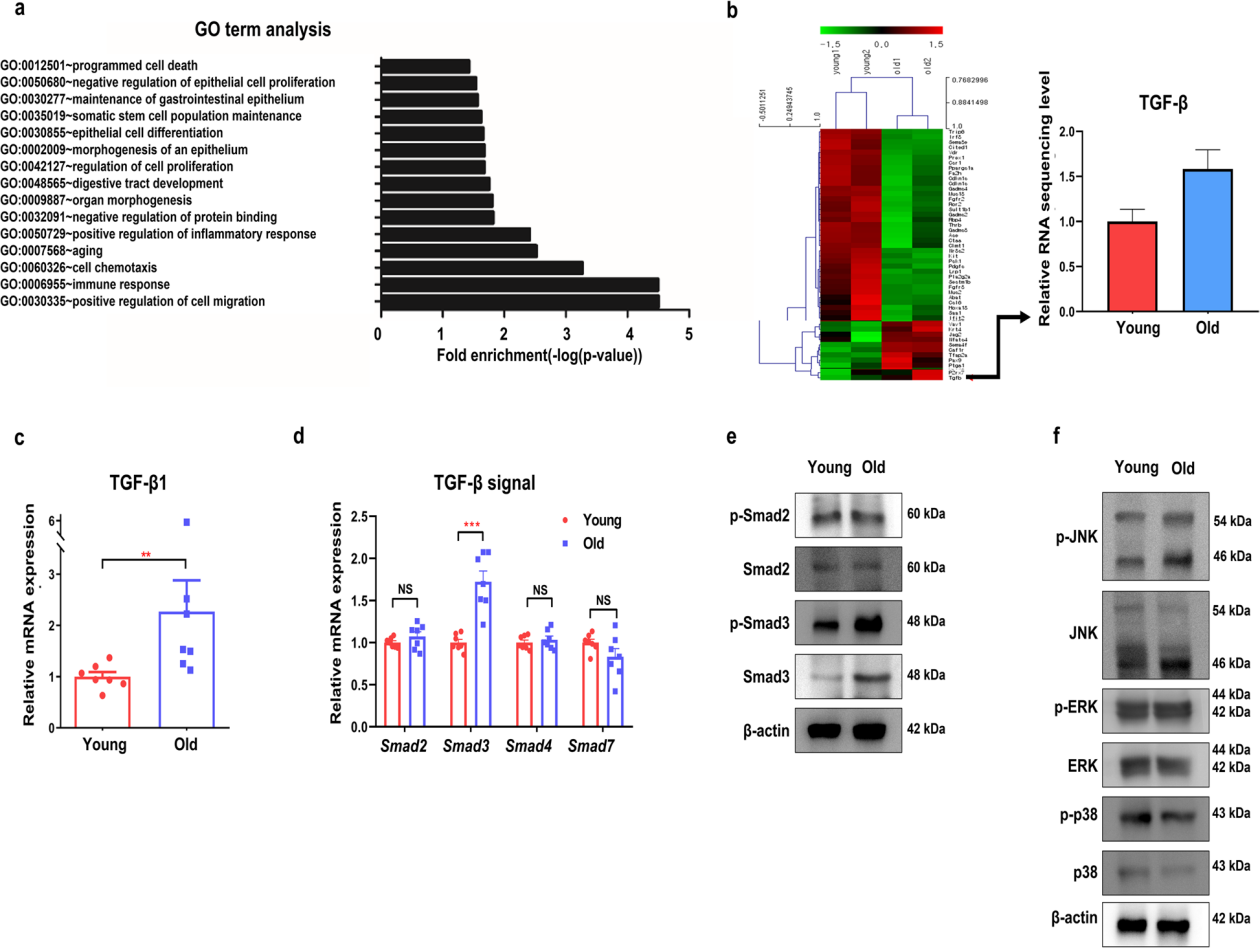
TGF-β-Smad3 signaling mediates the effects of aging on the colonic epithelial organoids. **a** GO terms analysis in the old-mouse organoids compared with the young-mouse organoids. Data are shown as the top 15 upregulated. **b** Heatmap displaying RNA-seq data of normalized and scaled genes on the young - and old-mouse organoids. The genes were selected from the results of the GO terms analysis and were manually grouped. The relative level of TGF-β expression in RNA-seq in colonic epithelial organoids from young and old mice is shown in the graph. **c** Relative levels of TGF-β1 mRNA expression in colonic epithelial organoids from young and old mice. Data are reported as the mean ± standard error of the mean (SEM) (*n* = 7). **d** Smad2, Smad3, Smad4, and Smad7 mRNA expression in the young- and old-mouse organoids. Data are normalized to the β-actin transcript and are reported as the mean ± SEM (*n* = 7). **e** Western blot showing the protein levels of p-Smad2, Smad2, p-Smad3, Smad3, and β-actin in colonic epithelial organoids from young and old mice. The images were cropped from the original blot image in Supplementary Fig. 4a. The blot was cut before hybridization with antibodies. **f** Western blot analysis of p-JNK, JNK, p-ERK, ERK, p-p38, p38, and β-actin in the young- and old-mouse organoids. The images were cropped from the original blot image in Supplementary Fig. 5a. The blot was cut before hybridization with antibodies. ^*^p < 0.05, ^**^p < 0.01, ^***^p < 0.001, ^****^p < 0.0001 by the Mann–Whitney t-test. TGF-β, transforming growth factor β; GO, gene ontology; p-JNK, phospho c-Jun N-termical kinases; JNK, c-Jun N-termical kinases; p-ERK, phospho-extracellular signal-regulated kinases; ERK, extracellular signal-regulated kinases

To identify the aging-associated gene network in the formation and growth of colonic organoids, we initially conducted transcriptome analysis using RNA sequencing on young- and old-mouse organoids. Through GO term analysis and DAVID functional annotation using the mRNA sequencing data, TGF-β and cell cycle associated genes were identified to be associated with the aging effect (Fig. 2a, Supplementary Fig. 1, Supplementary Table S6). Cell cycle-associated genes were related to the signaling pathway initiated from TGF-β in the PPI network (Supplementary Fig. 1). Old-mouse organoids exhibited significant upregulation of the TGF-β gene in a gene expression heat-map (Fig. 2b). When verified using qRT-PCR, TGF-β1 mRNA in old-mouse organoids was upregulated compared with that in young-mouse organoids (Fig. 2c, *p* = 0.004).

Next, we conducted experiments to evaluate which TGF-β-associated signaling pathway was related to our results. TGF-β signaling pathway has Smad-dependent (including Smad2/3) and non-Smad-dependent pathways, including JNK, p38, and ERK [14, 15]. When to assess a Smad-dependent signaling pathway, we confirmed that Smad3 expression in old-mouse organoids was upregulated compared with that in young-mouse organoids in qRT-PCR (*p* < 0.001). In addition, there was no difference between old- and young-mouse organoids in the expression of Smad2 (*p* = 0.456), Smad4 (*p* = 0.710), and Smad7 (*p* = 0.209) (Fig. 2d). However, no difference was observed in the bone morphogenetic proteins Bmp2, Bmp4, BmpR1A, and BmpR1B or Smad5 or Smad8 mRNA expression between the two groups (Supplementary Fig. 2a). The protein expression levels of Smad3 and p-Smad3 in the old-mouse organoids were markedly increased compared with those in the young-mouse organoids, while there was no difference in the expression levels of Smad2 and p-Smad2 between the two groups (Fig. 2e). We also evaluated a Smad-independent signaling in TGF-β-related pathways. The protein expression levels of p-JNK in the old-mouse organoids were slightly increased, and those of p38 and p-p38 were slightly decreased compared with those in young-mouse organoids. There was no difference in ERK or p-ERK expression (Fig. 2f). Taken together, our results show that TGF-β expression was markedly different between young- and old-mouse organoids, which was closely related to Smad3 among TGF-β-related signaling pathways.

### Cell cycle arrest through TGF-β-Smad3-p16^INK4a^ signaling is increased in old-mouse organoids

**Fig. 3:**
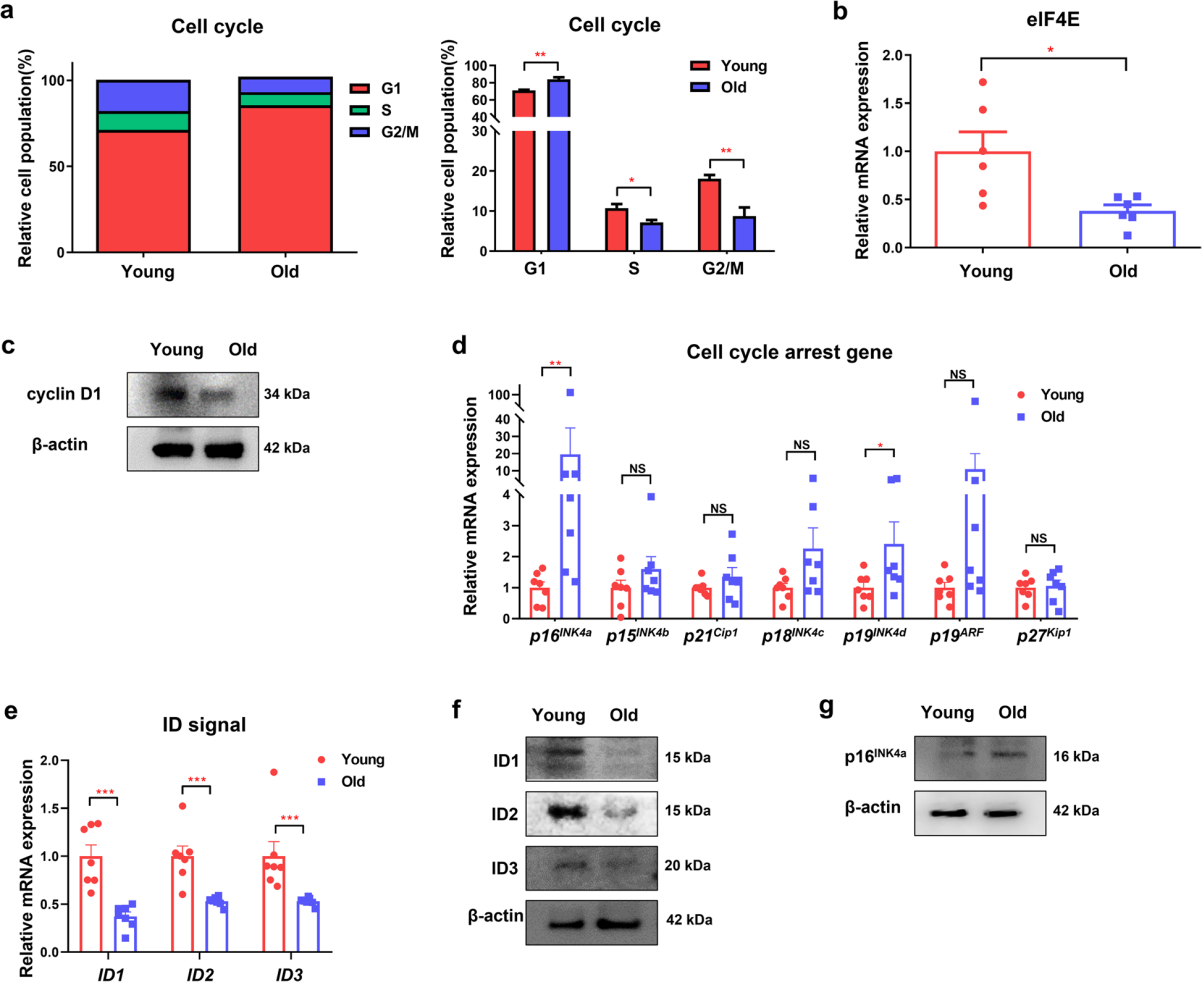
TGF-β-Smad3 signaling induces cell cycle arrest in the colonic epithelial organoids of old mice. **a** Cell cycle analysis through PI staining using flow cytometry (left) and quantitative measurement of the cell cycle phase in the young- and old-mouse organoids (rignt). **b** Relative levels of elF4E mRNA expression in colonic epithelial organoids from young and old mice. **c** Protein expression levels of cyclin D1 and β-actin in young- and old-mouse organoids. The images were cropped from the original blot image in Supplementary Fig. 6a. The blot was cut before hybridization with antibodies. **d** mRNA expression of cell cycle-regulated genes in young and old-mouse organoids. Data are shown as ± standard error of the mean (SEM) (*n* = 7). **e** ID1, ID2, and ID3 mRNA expression in young- and old-mouse organoids. Data are reported as the mean ± SEM (*n* = 7). **f** Protein expression levels of ID1, ID2, ID3, and β-actin in young- and old-mouse organoids. The images were cropped from the original blot image in Supplementary Fig. 6b. The blot was cut before hybridization with antibodies. **g** Protein expression levels of p-16^INK4a^ and β-actin in young- and old-mouse organoids. The images were cropped from the original blot image in Supplementary Fig. 6c. The blot was cut before hybridization with antibodies. ^*^p < 0.05, ^**^p < 0.01, ^***^p < 0.001,.^****^p < 0.0001 by the Mann–Whitney t-test. TGF-β, transforming growth factor β; ID1, inhitor of DNA binding 1; ID2, inhitor of DNA binding 2; ID3, inhitor of DNA binding 3

Next, we aimed to reveal how the different expression of TGF-β-Smad3 signaling impacts the formation and growth of colonic organoids between young and old mice. TGF-β1 has been reported to be closely related to intestinal homeostasis through modulation of proliferation, terminal differentiation, and stimulation of cellular migration in intestinal epithelial cells [16, 17]. Previous studies have demonstrated that Smad3 is associated with cell cycle arrest and growth inhibition [18–20]. In our study, in the cell cycle analysis, the G1 arrest cell population of the old-mouse organoids was significantly increased compared with that of the young-mouse organoids (Fig. 3a, G1 phase, *p* = 0.002, S phase, *p* = 0.026, G2/M phase, *p* = 0.004). The protein expression of cyclin D1 and mRNA expression of elF4e and Lgr5 were decreased in the old-mouse organoids, indicating that cell cycle arrest was augmented in this group (Fig. 3b, c, Supplementary Fig. 2b). These results suggest that the aging effect on colonic organoids is related to differences in cell cycle arrest between young and old mice.

**Fig. 4:**
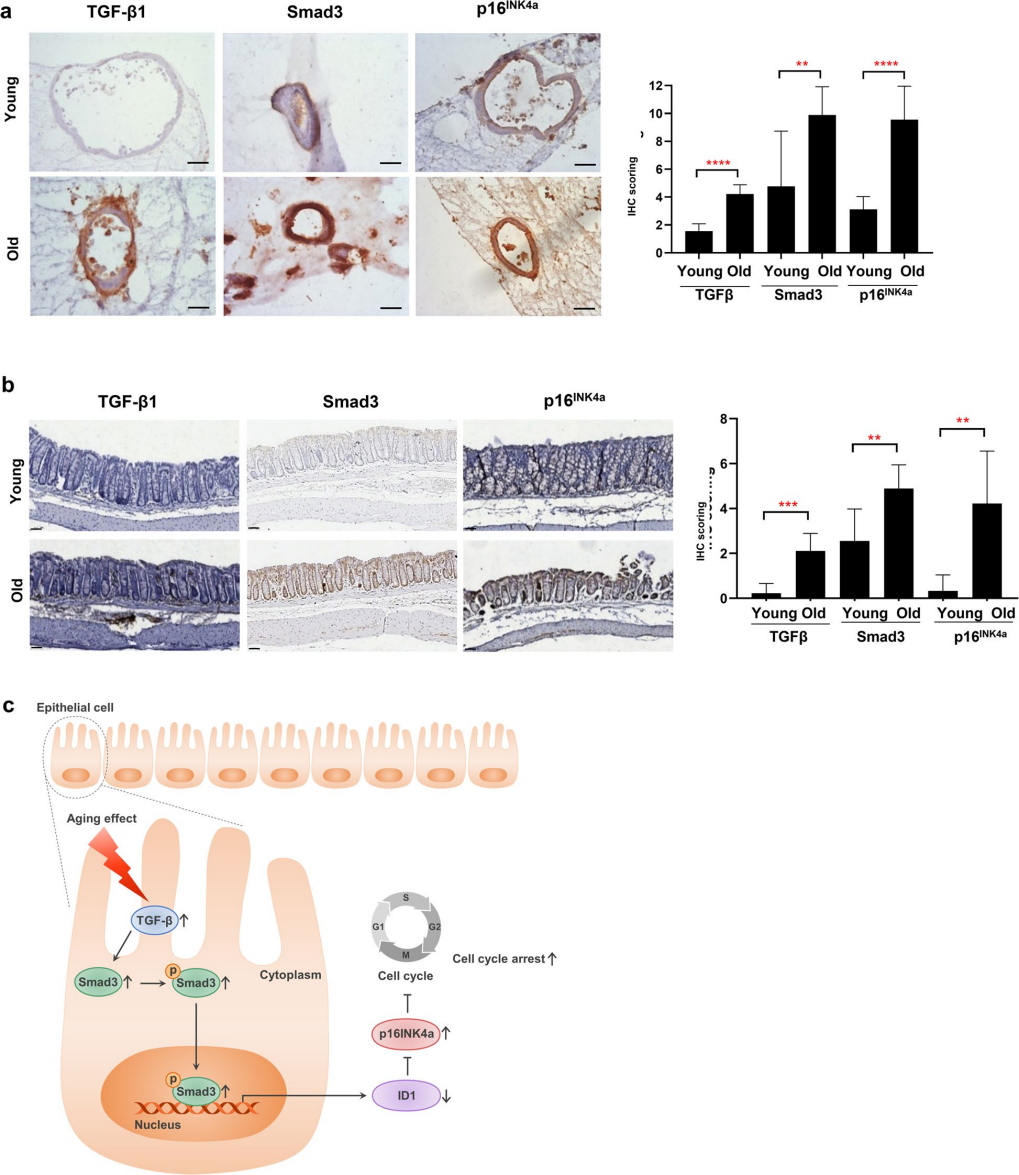
TGF-β1, Smad3, and p16^INK4a^ expression is increased in the colonic organoids and epithelium from old mice. **a** TGF-β1, Smad3, and p16^INK4a^ expression in the colonic epithelial organoids from young and old mice (left) and sum of immunohistochemistry (IHC) score (right) (3 organoids per mouse; 3 mice per group) (Scale bar = 100 μm). **b** TGF-β1, Smad3, and p16^INK4a^ expression in the colonic epithelium from young and old mice (left) and sum of IHC score (right) (3 mice per group, scale bar = 50 μm). **c** Schematic illustration of how aging impacts cell cycle arrest through TGF-β-Smad3 signaling in colonic epithelial cells. ^*^p < 0.05, ^**^p < 0.01, ^***^p < 0.001, ^****^p < 0.0001 by the Mann–Whitney t-test. TGF-β, transforming growth factor β; IHC, immunohistochemistry

Regarding Smad signaling and cell cycle arrest observed in our results, previous studies have also identified that the inhibitor of DNA binding (ID) signaling affects changes in the cell cycle and differentiation through Smad-dependent pathway [21–24]. When we assessed the ID downstream genes, the genes of cell cycle-dependent kinase inhibitors including p16^INK4a^ and p19^INK4d^ in the old-mouse organoids were significantly upregulated compared with that in the young-mouse organoids (*p* = 0.004, *p* = 0.038, respectively) (Fig. 3d). When evaluating the mRNA levels of the ID signal, ID1, ID2 and ID3 in the old-mouse organoids were significantly downregulated compared with those in the young-mouse organoids (*p* < 0.001) (Fig. 3e). In the Western blot analysis, ID1, ID2, and ID3 expression was also decreased in the old-mouse organoids (Fig. 3f). The protein expression of p16^INK4a^ was also increased in the old-mouse organoids (Fig. 3g). Subsequently, when comparing the protein expression in both organoid and colonic tissue, TGF-β1, Smad3, and p16^INK4a^ were significantly increased in both the organoids and colonic epithelium of the old mice compared with those of the young mice (Fig. 4a, b). Taken together, our results suggest that the reduced formation and growth of old-mouse organoids is attributed to increased cell cycle arrest with a high level of p16^INK4a^, which is affected by increased TGF-β-Smad3 signaling and decreased ID1 expression (Fig. 4c).

### Inhibition of TGF-β signaling increases the formation and growth of colonic organoids from old mice

**Fig. 5:**
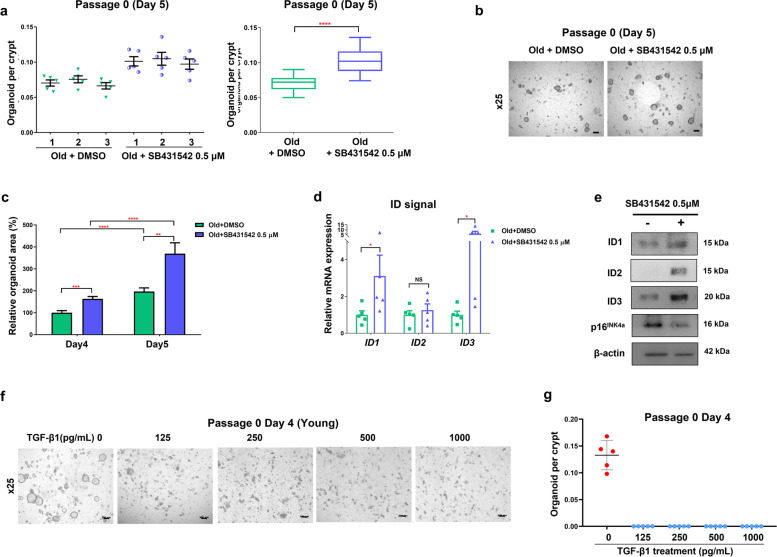
Formation and growth of old-mouse organoids is reversed by inhibition of TGF-β. **a** Old-mouse organoids treated with or without 0.5 μM SB431542, a TGF-β inhibitor. Organoids were cultured at a density of 500 crypts per well in the organoid culture (passage 0). Data are reported as the mean ± standard deviation (SD) of 15 wells (5 wells per mouse; 3 mice per group). **b** Representative images of the indicated groups on day 5 of passage 0 after seeding (Scale bar = 200 μm). **c** Relative change in the organoid area on days 4 and 5 of passage 0. The percentages are relative to the old-mouse organoids on day 4. Data are shown as the mean ± SD of 15 organoids (5 organoid per mouse; 3 mice per group). Data are reported as the mean ± SD. **d** ID1, ID2, and ID3 mRNA expression in old-mouse organoids with and without 0.5 μM SB431542. Data are reported as the mean ± SEM (standard error of the mean) (*n* = 5). **e** Protein expression levels of ID1, ID2, ID3, p16^INK4a^, and β-actin in old-mouse organoids with and without SB431542. The images were cropped from the original blot image in Supplementary Fig. 7a. The blot was cut before hybridization with antibodies. **f** Images of young-mouse organoids treated with or without various concentrations of TGFβ (0, 125, 250, 500, 1000 pg/mL) (Scale bar = 200 μm). The organoids were cultured at a density of 500 crypts per well in the organoid culture (passage 0) (n = 5 wells per mouse; n = 3 mice per group). **g** Number of organoid per crypt at passage 0 on day 4 ^*^p < 0.05, ^**^p < 0.01, ^***^p < 0.001,.^****^p < 0.0001 by the Mann–Whitney t-test. TGF-β1, transforming growth factor β1; ID1, inhitor of DNA binding 1; ID2, inhitor of DNA binding 2; ID3, inhitor of DNA binding 3

To further explore the role of TGF-β in mediating the formation and growth of colonic organoids, we cultured the old-mouse organoids treated with SB431542, a type I TGF-β receptor inhibitor (Fig. 5a, b, c, d, e), and the young mice-organoids treated with TGF-β1 (Fig. 5f, g). In the experiments of embedding 500 crypts in each well, the number of old-mouse organoids treated with SB431542 was significantly higher than that of old-mouse organoids treated with vehicle (0.101 ± 0.016 vs. 0.071 ± 0.010, per crypt, *p* < 0.0001) (Fig. 5a, b). With regard to the size of the organoids on day 4 of passage 0, the relative area of the old-mouse organoids treated with SB431542 was significantly larger than that of the old-mouse organoids with vehicle (163.1 ± 42.1% vs. 100.0 ± 37.0%, *p* < 0.001) (Fig. 5c). The organoid size in these two groups showed the same pattern on day 5 (369.0 ± 193.2% vs. 196.8 ± 63.3%, *p* < 0.01) (Fig. 5c). This result suggested that inhibition of TGF-β partially reversed the reduced formation and growth of colonic organoids from old mice.

Next, we evaluated whether TGF-β-Smad3 and ID signaling, which were previously identified to be associated with the formation and growth of colonic organoids, was changed in the TGF-β suppressed condition. In the old-mouse organoids treated with SB431542, the mRNA expression of ID1 (*p* = 0.016) and ID3 (*p* = 0.016) was significantly increased compared with that in vehicle-treated controls (Fig. 5d). The protein level of p16^INK4a^ in the old-mouse organoids treated with SB431542 was significantly decreased, and those of ID1, ID2 and ID3 were increased compared with vehicle-treated controls (Fig. 5e). In contrast, we also assessed whether TGF-β1 treatment inhibited the formation and growth of colonic organoids. Crypts were embedded with various concentrations of TGF-β1 treatment (0, 125, 250, 500, and 1000 pg/mL) in young-mouse organoids. No organoids were noted in all groups treated with various TGF-β1 concentrations on day 4 of passage 0, suggesting that organoid formation was markedly suppressed under TGF-β1 treatment (Fig. 5f, g). Based on these experiments, we suggest that the effect of aging on the formation and growth of colonic organoids is closely related to different expression of TGF-β-Smad3 signaling.

### The effect of aging on the formation of colonic epithelial organoids is not dependent on the culture condition

To assess whether the initial formation and growth of old and young-mouse organoids differ depending on the culture condition, we compared organoids in collagen type I and Matrigel condition at passage 0. In Matrigel condition, the number of the old-mouse organoids (0.088 ± 0.005) was significantly lower than that of the young-mouse organoids (0.126 ± 0.013, *p* < 0.001). When comparing organoid formation between the two conditions, the number of the old- and young-mouse organoids in Matrigel condition was significantly higher than that in collagen condition (*p* < 0.001) (Supplementary Fig. 3a, b). The organoid size between the young and old groups in Matrigel condition was significantly different (Supplementary Fig. 3c, d). In Matrigel condition, the relative organoid area in the old-mouse group (40.7 ± 22.9%) was significantly smaller than that in the young mouse group (100.0 ± 41.7%) on day 4 (*p* < 0.01). The organoid size in both groups showed the same pattern on day 5 (57.2 ± 27.6% in old mice vs. 232.4 ± 96.8% in young mice, *p* < 0.001) (Supplementary Fig. 3c). In terms of the organoid growth in Matrigel condition, the organoid area of young mice on day 5 was significantly larger than that on day 4 (*p* < 0.01), while there was no difference between the organoid area of old mice on day 4 and 5 (*p* = 0.195) (Supplementary Fig. 3c). The growth rate of colonic organoids in young mice in Matrigel condition was significantly faster than in old mice (*p* < 0.001) (Supplementary Fig. 3d). Taken together, the reduced forming ability and growth rate of colon organoids from old mice were also observed in Matrigel condition as in collagen condition. Additionally, similar to collagen condition, the mRNA levels of TGF-β, Smad3, and p16^INK4a^ in old-mouse organoids were significantly upregulated compared to those in young-mouse organoids in Matrigel condition (*p* < 0.01) (Supplementary Fig. 3e).

To assess the regenerative potential in colonic organoids, we assessed fetal markers, Ly6a/Sca-1, Clusterin, and TACSTD2. In both collagen and Matrigel condition, Ly6a/Sca-1 positive cell population in old-mouse organoids was significantly higher than young-mouse organoids (*p* < 0.001). Ly6a/Sca-1 positive cell population in both young- and old-mouse organoids in collagen condition was significantly higher than those in Matrigel condition (*p* < 0.001) (Supplementary Fig. 3f). The mRNA levels of Ly6a/Sca-1, Clusterin, and TACSTD2 in old-mouse organoids were upregulated compared with those of young-mouse organoids in both collagen and Matrigel conditions (Supplementary Fig. 3 g, h).

### Colonic epithelial recovery after tissue injury was decreased in old mice in acute DSS colitis

**Fig. 6:**
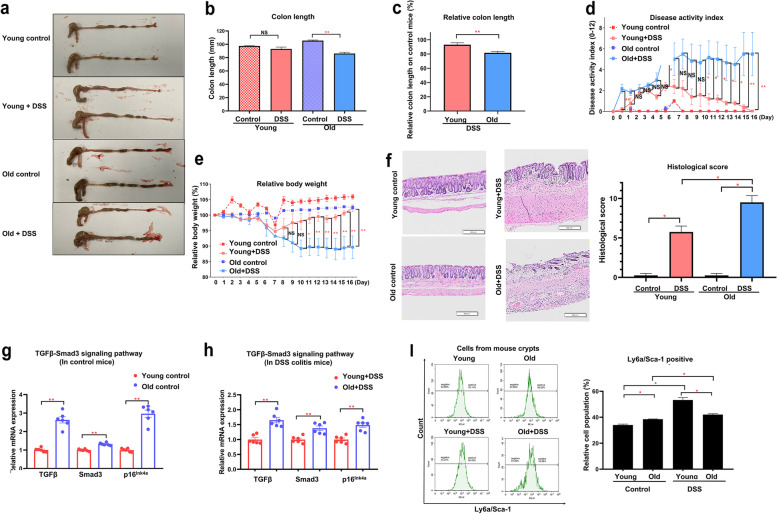
Colonic epithelial recovery after tissue injury was decreased in old mice with acute DSS colitis. **a** Representative images of the mouse colons after sacrifice. **b** The mouse-colon length after sacrifice (control mice, *n* = 4; DSS-treated mice, *n* = 6). **c** The relative colon length in the young+DSS and old+DSS group relative to the control groups (DSS-treated mice, *n* = 6). **d** The disease activity index (DAI) is composed of the change in body weight, diarrhea, and hematochezia (young+DSS vs. old+DSS group; control mice, n = 4; DSS-treated mice, *n* = 6). **e** Percentage change in the relative body weight (young+DSS vs. old+DSS group). **f** Hematoxylin and eosin (H&E) stained sections of colon and sum of histological colitis score (control mice, *n* = 4; DSS-treated mice, *n* = 4). **g, h** mRNA expression of TGF-β, Smad3, p16^INK4^ of the mouse colon crypts (*n* = 6) **i** Flow cytometric analysis of the cells from mouse colon crypts. Representative plots for Ly6a/Sca-1 positive cell population (control mice, *n* = 4; DSS-treated mice, *n* = 4). All data are reported as the mean ± SEM (standard error of the mean). ^*^p < 0.05, ^**^p < 0.01, ^***^p < 0.001, ^****^p < 0.0001

To compare the epithelial recovery after injury between young and old mice, we conducted acute DSS-colitis experiments. As a result, the final colon length in the DSS group was significantly shorter than the controls in old mice (86.170 ± 1.990 mm vs. 105.500 ± 0.866 mm, *p* < 0.01), while there was no difference between the two groups in young mice (92.990 ± 2.860 mm in DSS group vs. 97.500 ± 0.500 mm in controls, *p* = 0.105) (Fig. 6a, b). The colon length relative to the control groups was significantly shorter in the old+DSS group compared with the young+DSS group (81.7 ± 1.9% vs. 93.0 ± 2.9%, *p* < 0.01) (Fig. 6c). The old+DSS group showed a significantly higher disease activity index (DAI) (Fig. 6d) and lower relative body weight compared with the young+DSS group (Fig. 6e). Histologically, the old+DSS group (9.500 ± 0.866) showed a significantly higher colitis score compared with the young+DSS group (5.750 ± 0.750, *p* < 0.05) (Fig. 6f). In the mouse colon tissue, mRNA levels of TGF-β, Smad3, and p16 ^INK4a^ in the old controls and old+DSS group were upregulated compared with the young controls and young+DSS group, respectively (Fig. 6g, h). In flow cytometry analysis of cells from mouse colon crypts, Ly6a/Sca-1 positive population of the old controls was significantly higher than that of the young controls (38.5 ± 0.2% vs. 34.0 ± 0.7%, *p* < 0.05), while that of the old+DSS group was lower than the young+DSS group (42.1 ± 0.8% vs. 53.3 ± 2.0%, *p* < 0.05) (Fig. 6i). Taken together, the colonic epithelial regeneration after tissue injury in old mice was significantly decreased compared with young mice. This phenomenon may be associated with an upregulated expression of TGF-β, Smad3, and p16^INK4a^ and downregulated expression of fetal marker, Ly6a/Sca-1 in old mice.

### The formation and growth of colonic epithelial organoids were reduced in old mice with acute DSS colitis

**Fig. 7:**
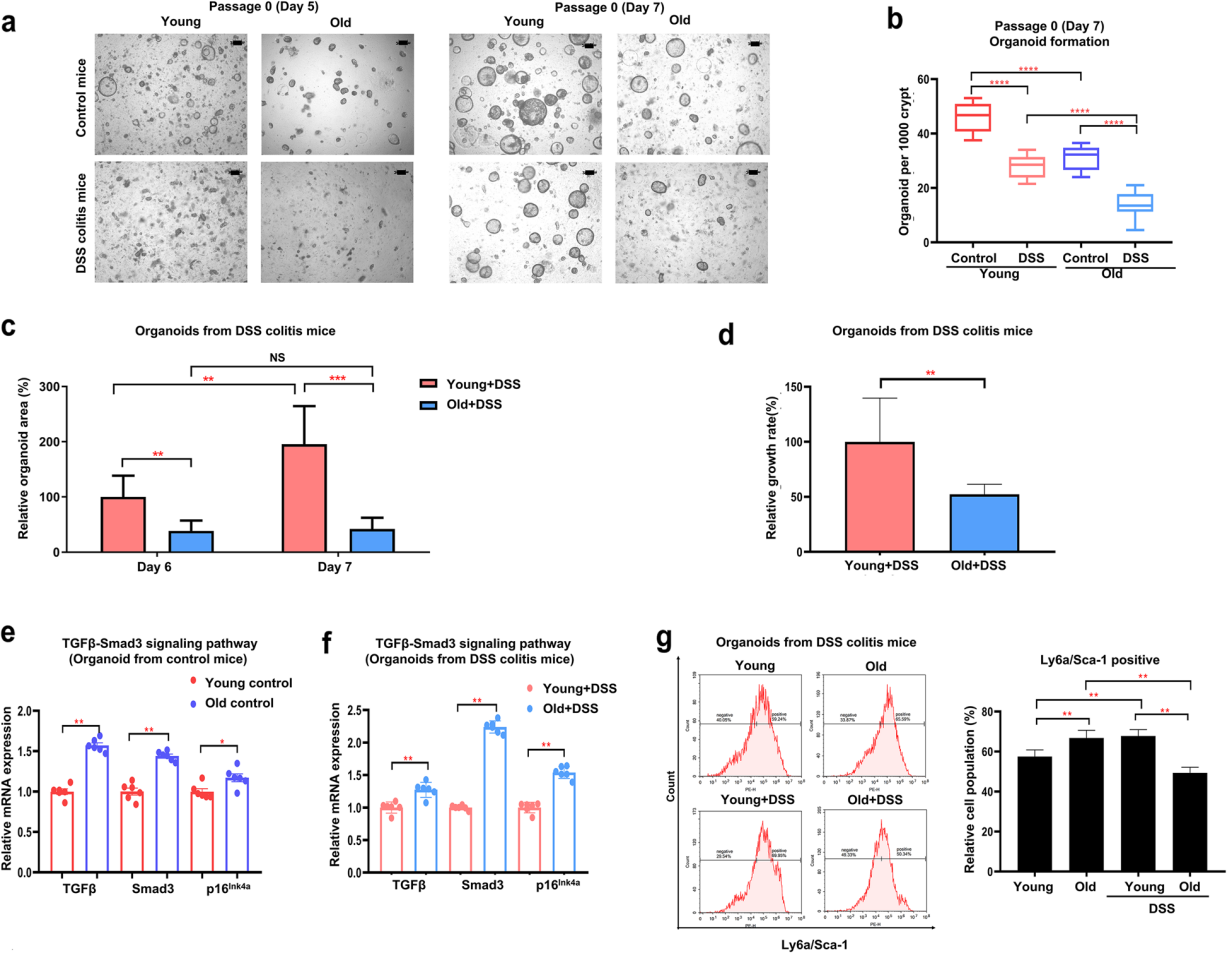
The formation and growth of colonic epithelial organoids were reduced in old mice with acute DSS-colitis. **a** Representative images of organoids on day 5 and 7 of passage 0 (organoids from young and old control and DSS-colitis mice sacrificed at 16 days after DSS initiation). **b** The number of organoids was cultured at a density of 2000 crypts per well. Data are reported as the mean ± standard deviation (SD) of 10 wells. **c** The relative organoid area from young and old DSS-colitis mice at days 6 and 7. The represented percentage is relative to the organoids of young DSS-colitis mice on day 6. Data are reported as the mean ± SD of 8 organoids. **d** The growth rate of the organoids from young and old DSS-colitis mice from day 6 to day 7. Data are reported as the mean ± SD of 8 organoids. **e, f** mRNA expression of TGF-β, Smad3, p16^INK4a^ of the organoids from young and old control or DSS-colitis mice. Data are reported as the mean ± SD (*n* = 6). **g** Flow cytometric analysis of organoid cells (organoids from young and old control and DSS-colitis mice). Representative plots for Ly6a/Sca-1 positive cell population. Data are reported as the mean ± SD (n = 5). ^*^p < 0.05, ^**^p < 0.01, ^***^p < 0.001,.^****^p < 0.0001

To compare the epithelial recovery upon tissue injury between young and old mice in organoid models, we cultured the organoids from DSS-colitis mice sacrificed at day 16 in the collagen type I condition. The organoids from old DSS-colitis mice at day 5 of passage 0 were hardly formed, making it difficult to measure their number. (Fig. 7a). The number of organoids from old DSS-colitis mice (13.900 ± 4.683/1000 crypts) was significantly lower than that from young DSS-colitis mice (27.950 ± 4.180/1000 crypts, *p* < 0.0001) at day 7 of passage 0. The number of organoids from old and young DSS-colitis mice was significantly lower than that from old and young controls (*p* < 0.0001), respectively (Fig. 7b). The organoid size between the young and old DSS-colitis mice also differed (Fig. 7c, d). The relative organoid area from old DSS-colitis mice was significantly smaller than that from young DSS-colitis mice on day 6 (38.7 ± 18.8% vs. 100.0 ± 38.7%, *p* < 0.01) and day 7 (42.2 ± 20.2% vs. 195.7 ± 68.9%, *p* < 0.001) (Fig. 7c). The growth rate of colonic organoids from day 6 to 7 in young DSS-colitis mice was significantly faster than that in old DSS-colitis mice (*p* < 0.01) (Fig. 7d). The mRNA levels of TGF-β, Smad3, and p16^INK4a^ in the old control and old + DSS mouse organoids were upregulated compared with the young control and young + DSS mouse organoids, respectively (Fig. 7e, f). In flow cytometry of organoid cells, Ly6a/Sca-1 positive population in the old controls was significantly higher than the young controls (66.75 ± 3.88% vs. 57.53 ± 3.30%, *p* < 0.01). Ly6a/Sca-1 positive population in the old + DSS group was lower than the young + DSS group (49.38 ± 2.81% vs. 67.77 ± 3.19%, *p* < 0.01) (Fig. 7g). These results showed that aging affected the epithelial regeneration after injury in organoid models as well as in vivo colitis experiments.

## Discussion

In this study, we investigated age-related phenotypes in the colonic epithelium and identified major related signaling pathways using an organoid model. When evaluating the effect of aging on the intestinal epithelium, the formation ability and growth rate of colonic epithelial organoids in old mice decreased compared with those in young mice. Similar to our results, previous studies have also reported that old-mouse organoids formed through the aging effect exhibited a smaller size and slower growth rate [25–28], suggesting that organoids from aged mice were significantly deficient in intestinal stem cell (ISC) function and regenerative potential [25, 26]. The aging process is characterized by a decreased self-renewal capacity of ISCs, a disproportionate differentiation of progenitors, and a reduced self-repair function of tissue [5]. Several studies have reported that aging affects the homeostasis of the intestinal epithelium through various signaling pathways [3, 25, 26, 29]. Among them, WNT signaling in the intestinal epithelium has been actively studied and is important for tissue homeostasis in young mice [30]. Some researchers have identified that the impaired regenerative capacity of ISCs upon aging depends on a decrease in the canonical WNT signaling of ISCs and their niche [25, 26]. Accordingly, reactivation of canonical WNT signaling restores ISC function in aged organoids [25].

In the current study, to identify the genes associated with the effect of aging on colonic organoids, we conducted transcriptome analysis and eventually identified TGF-β- and cell cycle-related genes. In addition, we confirmed that TGF-β expression in mRNA and protein levels was significantly increased in old-mouse organoids compared with those in young-mouse organoids. TGF-β plays an important role in gut development as a key signaling pathway through interaction with WNT signaling [31]. TGF-β1 is also related to intestinal homeostasis through modulation of proliferation, terminal differentiation, and stimulation of cellular migration in intestinal epithelial cells [16, 17]. In the study by Yamada et al. [32]. that evaluated the effects of TGF-β1 on the functions of intestinal epithelial cells and its related signaling, TGF-β1 was produced and secreted by IEC-6 cells, rat intestinal epithelial cells, in an autocrine fashion. Based on our results and those of previous studies, we initially hypothesized that the different phenotype (forming ability and growth rate) between young- and old-mouse organoids originates from differential expression of TGF-β. To verify this hypothesis, we conducted further experiments and demonstrated that the forming ability and growth rate in old-mouse organoids recovered with TGF-β inhibition (SB431542 treatment), while young-mouse organoids did not form with TGF-β treatment.

TGF-β signaling acts through Smad-dependent or non-Smad-dependent pathways, and recent evidence has suggested that Smad2 and Smad3 have different and separate roles in TGF-β signaling [14, 33]. One study reported that TGF-β-related cytostatic signals depend on the Smad3- but not the Smad2-dependent pathway and that Smad3 may play a more pivotal role than Smad2 in TGF-β-related cell cycle arrest [34]. The other study using IEC-6 cells also showed that TGF-β1 suppressed epithelial proliferation through a Smad3-dependent pathway [32]. In this study, we conducted further experiments to identify which signaling pathway related to TGF-β is associated with the different organoid phenotype between young and old mice. Regarding the Smad-dependent pathway, the old-mouse organoids showed significantly increased expression of Smad3 in both mRNA and protein level, but not Smad2. However, with regard to the Smad-independent pathway, no difference was observed in the BMP expression between the two groups.

In the present study, we also found decreased expression of the ID family and increased expression of p16^INK4a^ in the old mice, which were associated with increased cell cycle arrest and reduced formation and growth of old-mouse organoids. These results of our experiments are in accordance with those of previous studies. One study demonstrated that the inhibition of ID1 by TGF-β1 was recovered by treatment of Smad3 siRNA but not Smad2 siRNA in LoVo cells, which suggested that TGF-β1 inhibited ID1 expression through a Smad3-depedent pathway [35]. ID proteins were identified to act as a positive regulator in cell proliferation and a negative regulator in cell differentiation [36, 37]. With regard to p16^INK4a^, detailed quantification showed that p16^INK4a^ expression increases with age in all mammalian species [38], which makes p16^INK4a^ one of the most potent aging biomarkers. In a study using a transgenic mouse model with the removal of p16^INK4a^ senescent cells, the deletion of p16^INK4a^-expressing cells selectively decreased age-related pathologies in tissues, such as skeletal muscle, eye, and adipose tissue [39]. In a variety of stress conditions, p16 expression suppresses inappropriate cell division, and an induction of p16^INK4a^ causes cellular senescence, namely irreversible cell cycle arrest [40]. Among several signaling pathways, TGF-β-Smad3 was reported to be related to p16^INK4a^ expression in aged tissues. Age-related increases in local TGF-β production in muscle satellite cells activate SMAD3, which binds to the p16^INK4a^ promoter and initiates gene transcription [41]. In addition, p16^INK4a^ may be also suppressed by ID1. In cellular senescence, p16^INK4a^ is induced by E-26 transformation-specific 1 (ETS1), ETS2, and E47 [42]. ID1 inhibited this process by preventing the binding of ETS1, ETS2, and E47 on the p16^INK4a^ promoter [42, 43]. Another study demonstrated that embryonic fibroblasts from ID1-null mice show premature senescence and have high levels of p16^INK4a^ expression [44]. Taken together, in our study, the reduced formation and growth of old-mouse organoids were closely related to increased cell cycle arrest with high levels of p16^INK4a^ expression, which was influenced by both TGF-β-Smad3 and ID1.

Next, we also evaluated whether aging also impacts the colonic epithelial regeneration after tissue injury. In this study, the colonic recovery in the old mice was significantly decreased compared with the young mice during in vivo DSS-colitis experiments, which was also reproduced in an ex vivo organoid model. The expression of TGF-β, Smad3, and p16^INK4a^ in the DSS-treated old mice was significantly lower than in DSS-treated young mice in colon tissue and organoids, which suggests that the regenerative potential after injury is suppressed by cell cycle arrest through TGF-β-Smad3 signaling in the aged colon.

Additionally, our results also showed that the effect of aging on the formation and growth of colonic organoids is not dependent on the culture condition. Among organoid culture systems, collagen type I is suitable for exhibiting the regeneration and inflammation of epithelium [45, 46]. Collagen type I significantly influenced the fetal-like reprogramming of intestinal epithelium during inflammation [45]. Therefore, to assess whether the organoid phenotype is different between in collagen and Matrigel conditions, we cultured and compared organoids in both conditions. Consequently, the reduced forming ability and growth rate of colon organoids from old mice were also observed in Matrigel condition as in collagen condition.

On the other hand, several results of our study need to be considered. First, in this study, as the passage of colonic organoids increased, the difference in the organoid forming ability between the young and old mice became smaller. This phenomenon can be explained by the effect of niche, namely, a complex microenvironment surrounding ISCs. The niche consists of immune cells, fibroblasts, muscle cells, endothelial cells, and adipocytes, and the signals from the niche regulate ISC behaviors [47]. In our results, the effect of niche disappeared in the colonic organoids in later passages. However, expression of TGF-β, Smad3, and p16^INK4a^ was also increased in the colonic tissue as well as the organoids, suggesting that the niche partially influenced it. Second, the expression of JNK and p-JNK in Smad-independent signaling was slightly increased in the old-mouse organoids. Further study is needed to evaluate the association of other TGF-β signal pathways with age-related phenotypes in the colonic epithelium.

## Conclusions

In the present study, we found that aging reduces the forming ability and growth rate of colonic epithelial organoids, which is associated with an increased TGF-β expression. Among TGF-β-related pathways, this age-specific phenotype of old-mouse organoids is attributed to an increased cell cycle arrest with high levels of p16^INK4a^, which are affected by an increased TGF-β-Smad3 signaling and decreased ID1 expression. We verified this by conducting experiments using TGF-β inhibition on old-mouse organoids and TGF-β1 treatment on young-mouse organoids. Additionally, aging reduces an epithelial regeneration after tissue injury, which is affected by cell cycle arrest through TGF-β-Smad3 signaling. Our research can provide new information about the novel approaches for management or treatment of aging-related intestinal diseases that are rapidly on the rise in recent years.

## Supplementary Information

**Additional file 1:** **Supplementary Fig. 1.** Protein–protein interaction network of differentially expressed genes. **Supplementary Fig. 2.** Quantitative RT-PCR analysis of BMP pathway and stem cell signature gene levels in young- and old-mouse organoids. **Supplementary Fig. 3.** The effect of aging on the formation of colonic epithelial organoids is not dependent on the culture condition. **Supplementary Fig. 4.** Original blots of Western blot. **Supplementary Fig. 5.** Original blots of Western blot. **Supplementary Fig. 6.** Original blots of Western blot. **Supplementary Fig. 7.** Original blots of Western blot.

**Additional file 2.** 

## Data Availability

Not applicable.

## References

[1] Lopez-Otin C, Blasco MA, Partridge L, Serrano M, Kroemer G (2013). The hallmarks of aging. Cell.

[2] He S, Sharpless NE (2017). Senescence in Health and Disease. Cell.

[3] Tominaga K, Suzuki HI (2019). TGF-beta signaling in cellular senescence and aging-related pathology. Int J Mol Sci.

[4] Hu JL, Todhunter ME, LaBarge MA, Gartner ZJ (2018). Opportunities for organoids as new models of aging. J Cell Biol.

[5] Funk MC, Zhou J, Boutros M (2020). Ageing, metabolism and the intestine. EMBO Rep.

[6] Li M, Xiao ZQ, Chen ZC, Li JL, Li C, Zhang PF (2007). Proteomic analysis of the aging-related proteins in human normal colon epithelial tissue. J Biochem Mol Biol.

[7] Okumura R, Takeda K (2017). Roles of intestinal epithelial cells in the maintenance of gut homeostasis. Exp Mol Med.

[8] Tran L, Greenwood-Van MB (2013). Age-associated remodeling of the intestinal epithelial barrier. J Gerontol A Biol Sci Med Sci.

[9] Holt PR (2007). Intestinal malabsorption in the elderly. Dig Dis.

[10] Dukowicz AC, Lacy BE, Levine GM (2007). Small intestinal bacterial overgrowth: a comprehensive review. Gastroenterol Hepatol (N Y).

[11] Edwards BK, Noone AM, Mariotto AB, Simard EP, Boscoe FP, Henley SJ (2014). Annual Report to the Nation on the status of cancer, 1975–2010, featuring prevalence of comorbidity and impact on survival among persons with lung, colorectal, breast, or prostate cancer. Cancer.

[12] Radtke F, Clevers H (2005). Self-renewal and cancer of the gut: two sides of a coin. Science.

[13] Miyoshi H, Stappenbeck TS (2013). In vitro expansion and genetic modification of gastrointestinal stem cells in spheroid culture. Nat Protoc.

[14] Moustakas A, Heldin CH (2005). Non-Smad TGF-beta signals. J Cell Sci.

[15] Derynck R, Zhang YE (2003). Smad-dependent and Smad-independent pathways in TGF-beta family signalling. Nature.

[16] Barnard JA, Beauchamp RD, Coffey RJ, Moses HL (1989). Regulation of intestinal epithelial cell growth by transforming growth factor type beta. Proc Natl Acad Sci U S A.

[17] Sturm A, Dignass AU (2008). Epithelial restitution and wound healing in inflammatory bowel disease. World J Gastroenterol.

[18] Granados-Aparici S, Hardy K, Franks S, Sharum IB, Waite SL, Fenwick MA (2019). SMAD3 directly regulates cell cycle genes to maintain arrest in granulosa cells of mouse primordial follicles. Sci Rep.

[19] Mithani SK, Balch GC, Shiou S-R, Whitehead RH, Datta PK, Beauchamp RD (2004). Smad3 has a critical role in TGF-β-mediated growth inhibition and apoptosis in colonic epithelial cells. J Surg Res.

[20] Park SJ, Yang SW, Kim BC (2016). Transforming growth factor-beta1 induces cell cycle arrest by activating atypical cyclin-dependent kinase 5 through up-regulation of Smad3-dependent p35 expression in human MCF10A mammary epithelial cells. Biochem Biophys Res Commun.

[21] Qi Z, Li Y, Zhao B, Xu C, Liu Y, Li H (2017). BMP restricts stemness of intestinal Lgr5(+) stem cells by directly suppressing their signature genes. Nat Commun.

[22] Kowanetz M, Valcourt U, Bergstrom R, Heldin CH, Moustakas A (2004). Id2 and Id3 define the potency of cell proliferation and differentiation responses to transforming growth factor beta and bone morphogenetic protein. Mol Cell Biol.

[23] Ling MT, Wang X, Tsao SW, Wong YC (2002). Down-regulation of Id-1 expression is associated with TGFβ1-induced growth arrest in prostate epithelial cells. Biochimica et Biophys Acta (BBA) - Gen Sub.

[24] Zhang Y, Alexander PB, Wang X-F (2017). TGF-β family signaling in the control of cell proliferation and survival. Cold Spring Harb Perspect Biol.

[25] Nalapareddy K, Nattamai KJ, Kumar RS, Karns R, Wikenheiser-Brokamp KA, Sampson LL (2017). Canonical Wnt signaling ameliorates aging of intestinal stem cells. Cell Rep.

[26] Cui H, Tang D, Garside GB, Zeng T, Wang Y, Tao Z (2019). Wnt signaling mediates the aging-induced differentiation impairment of intestinal stem cells. Stem Cell Rev Rep.

[27] Moorefield EC, Andres SF, Blue RE, Van Landeghem L, Mah AT, Santoro MA (2017). Aging effects on intestinal homeostasis associated with expansion and dysfunction of intestinal epithelial stem cells. Aging (Albany NY).

[28] Uchida R, Saito Y, Nogami K, Kajiyama Y, Suzuki Y, Kawase Y (2018). Epigenetic silencing of Lgr5 induces senescence of intestinal epithelial organoids during the process of aging. npj Aging and Mechanism Disease.

[29] Pollina EA, Brunet A (2011). Epigenetic regulation of aging stem cells. Oncogene.

[30] Pinto D, Gregorieff A, Begthel H, Clevers H (2003). Canonical Wnt signals are essential for homeostasis of the intestinal epithelium. Genes Dev.

[31] Moses HL, Yang EY, Pietenpol JA (1991). Regulation of epithelial proliferation by TGF-beta. Ciba Found Symp.

[32] Yamada Y, Mashima H, Sakai T, Matsuhashi T, Jin M, Ohnishi H (2013). Functional roles of TGF-beta1 in intestinal epithelial cells through Smad-dependent and non-Smad pathways. Dig Dis Sci.

[33] Moustakas A, Heldin CH (2009). The regulation of TGFbeta signal transduction. Development.

[34] Kim SG, Kim HA, Jong HS, Park JH, Kim NK, Hong SH (2005). The endogenous ratio of Smad2 and Smad3 influences the cytostatic function of Smad3. Mol Biol Cell.

[35] Song H, Guo B, Zhang J, Song C (2010). Transforming growth factor-beta suppressed Id-1 Expression in a smad3-dependent manner in LoVo cells. Anat Rec (Hoboken).

[36] Norton JD, Deed RW, Craggs G, Sablitzky F (1998). Id helix-loop-helix proteins in cell growth and differentiation. Trends Cell Biol.

[37] Yokota Y, Mori S (2002). Role of Id family proteins in growth control. J Cell Physiol.

[38] Kim WY, Sharpless NE (2006). The regulation of INK4/ARF in cancer and aging. Cell.

[39] Baker DJ, Wijshake T, Tchkonia T, LeBrasseur NK, Childs BG, van de Sluis B (2011). Clearance of p16Ink4a-positive senescent cells delays ageing-associated disorders. Nature.

[40] LaPak KM, Burd CE (2014). The molecular balancing act of p16(INK4a) in cancer and aging. Mol Cancer Res.

[41] Carlson ME, Hsu M, Conboy IM (2008). Imbalance between pSmad3 and Notch induces CDK inhibitors in old muscle stem cells. Nature.

[42] Ohtani N, Zebedee Z, Huot TJ, Stinson JA, Sugimoto M, Ohashi Y (2001). Opposing effects of Ets and Id proteins on p16INK4a expression during cellular senescence. Nature.

[43] Zheng W, Wang H, Xue L, Zhang Z, Tong T (2004). Regulation of cellular senescence and p16(INK4a) expression by Id1 and E47 proteins in human diploid fibroblast. J Biol Chem.

[44] Alani RM, Young AZ, Shifflett CB (2001). Id1 regulation of cellular senescence through transcriptional repression of p16/Ink4a. Proc Natl Acad Sci U S A.

[45] Kobayashi S, Ogasawara N, Watanabe S, Yoneyama Y, Kirino S, Hiraguri Y (2022). Collagen type I-mediated mechanotransduction controls epithelial cell fate conversion during intestinal inflammation. Inflamm Regen.

[46] Yui S, Azzolin L, Maimets M, Pedersen MT, Fordham RP, Hansen SL (2018). YAP/TAZ-dependent reprogramming of colonic epithelium links ECM remodeling to tissue regeneration. Cell Stem Cell.

[47] Ollivier A, Mahe MM, Guasch G (2021). Modeling gastrointestinal diseases using organoids to understand healing and regenerative processes. Cells.

