# Supplementary material for: Effect of aging on the formation and growth of colonic epithelial organoids by changes in cell cycle arrest through TGF-β-Smad3 signaling
Source: Inflamm Regen. 2023 Jul 13;43:35. doi: 10.1186/s41232-023-00282-6 (PMC10339613; doi:10.1186/s41232-023-00282-6)
Supplement: Supplementary file 1 — Additional file 1: Supplementary Fig. 1. Protein–protein interaction network of differentially expressed genes. Supplementary Fig. 2. Quantitative RT-PCR analysis of BMP pathway and stem cell signature gene levels in young- and old-mouse organoids. Supplementary Fig. 3. The effect of aging on the formation of colonic epithelial organoids is not dependent on the culture condition. Supplementary Fig. 4. Original blots of Western blot. Supplementary Fig. 5. Original blots of Western blot. Supplementary Fig. 6. Original blots of Western blot. Supplementary Fig. 7. Original blots of Western blot. [file 41232_2023_282_MOESM1_ESM.pdf]

Supplementary Figures for

"Effect of aging on the formation and growth of colonic epithelial organoids by changes in cell cycle arrest through TGF-β-Smad3 signaling"

Min Kyoung Jo[1,2], Chang Mo Moon[1,2*], Hyeon-Jeong Jeon[1,2], Yerim Han[1,2], Eun Sook Lee[1,2], Ji-Hee Kwon[3], Kyung-Min Yang[4], Young-Ho Ahn[2,5], Seong-Eun Kim[1], Sung-Ae Jung[1], and Tae Il Kim[3*]

[1]Department of Internal Medicine, College of Medicine, Ewha Womans University, Seoul, Republic of Korea
[2]Inflammation-Cancer Microenvironment Research Center, College of Medicine, Ewha Womans University, Seoul, Republic of Korea
[3]Department of Internal Medicine, Yonsei University College of Medicine, Seoul, Republic of Korea
[4]Medpacto Inc., Seoul, Republic of Korea.
[5]Department of Molecular Medicine, College of Medicine, Ewha Womans University, Seoul, Republic of Korea

[*]Chang Mo Moon and Tae Il Kim contributed equally to this study as co-corresponding authors.

Correspondence

Chang Mo Moon, MD, PhD
Department of Internal Medicine and Inflammation-Cancer Microenvironment Research Center
College of Medicine, Ewha Womans University
1071 Anyangcheon-ro, Yangcheon-gu, Seoul, 07985, Republic of Korea
Phone: +82-2-2650-2945, Fax: +82-2-2650-5936, E-mail: mooncm27@ewha.ac.kr

Tae Il Kim, MD, PhD
Division of Gastroenterology and Department of Internal Medicine
Yonsei University College of Medicine
50-1 Yonsei-ro, Seodaemun-gu, Seoul 03722, Republic of Korea
Phone: +82-2-2228-1965, Fax: +82-2-393-6884, E-mail: taeilkim@yuhs.ac

Supplementary Figures

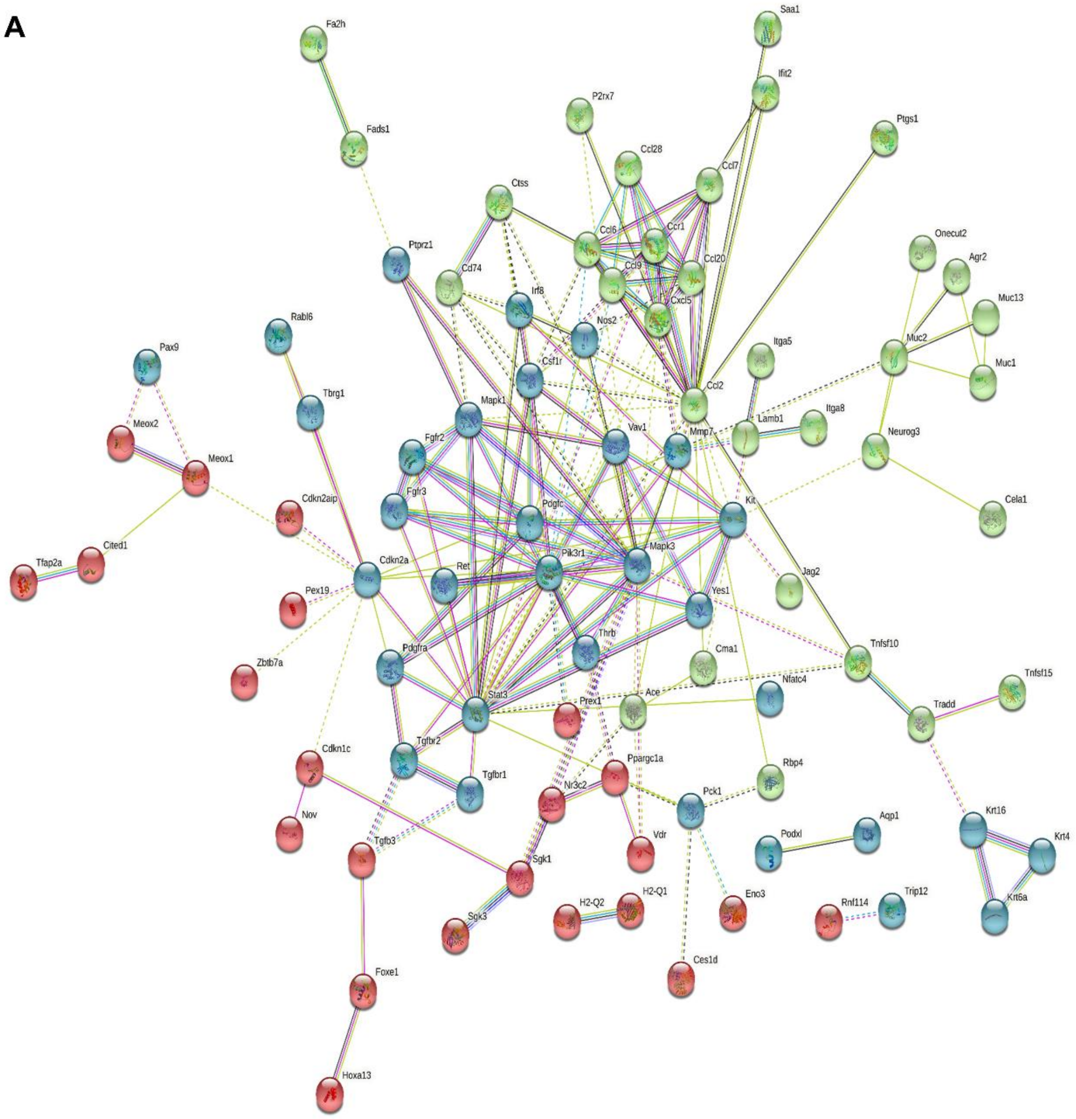

Supplementary Fig. 1 Protein–protein interaction network of differentially expressed genes.
GO terms enriched in differentially expressed genes (DEGs) upregulated from STRING analysis. The enriched pathways for DEGs in the protein-protein interaction (PPI) network.

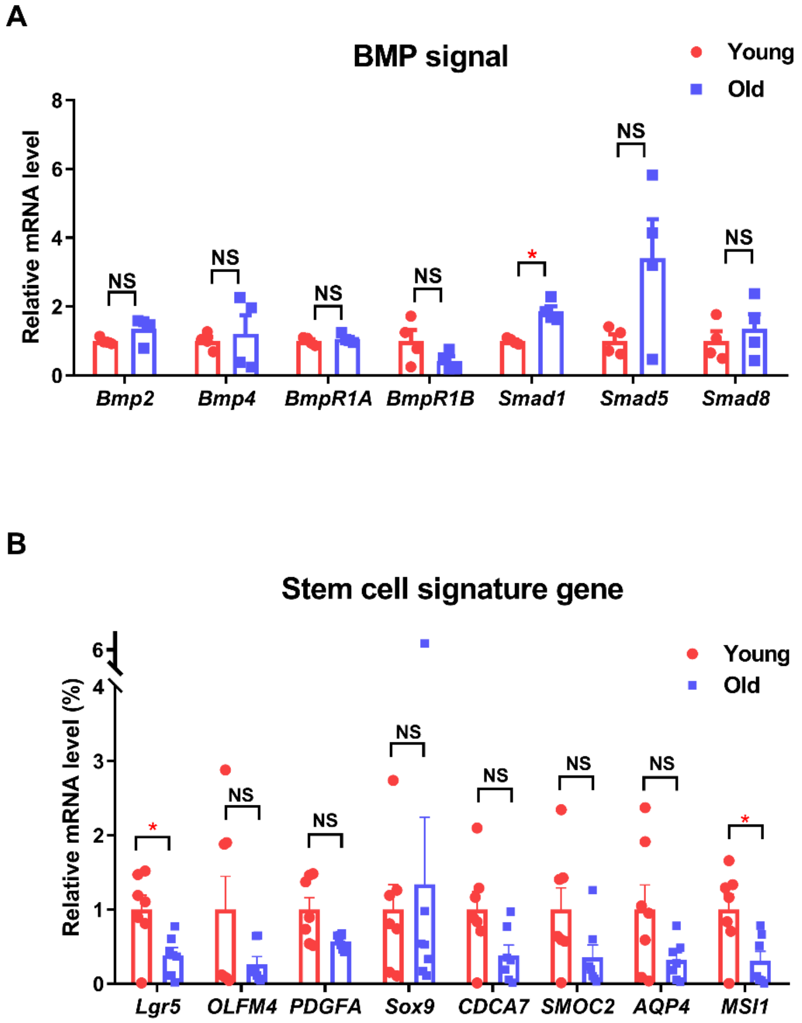

Supplementary Fig. 2 Quantitative RT-PCR analysis of BMP pathway and stem cell signature gene levels in young- and old-mouse organoids.

(**A**) Quantitative RT-PCR analysis of Bmp2, Bmp4, BmpR1A, BmpR1B, Smad1, Smad5, and Smad8 (BMP pathway) gene expression in young- and old-mouse organoids. Data are normalized to the β-actin transcript and are reported as the mean ± SEM (n = 4). (**B**) Lgr5, OLFM4, PDGFA, Sox9, CDCA7, SMOC2, AQP4 and MSI1 expression in the young- and old-mouse organoids. Data are normalized to the β-actin transcript and are reported as the mean ± SEM (n = 7). **p* <0.05, ***p* <0.01, ****p* <0.001, *****p* <0.0001 by Mann-Whitney t-test.

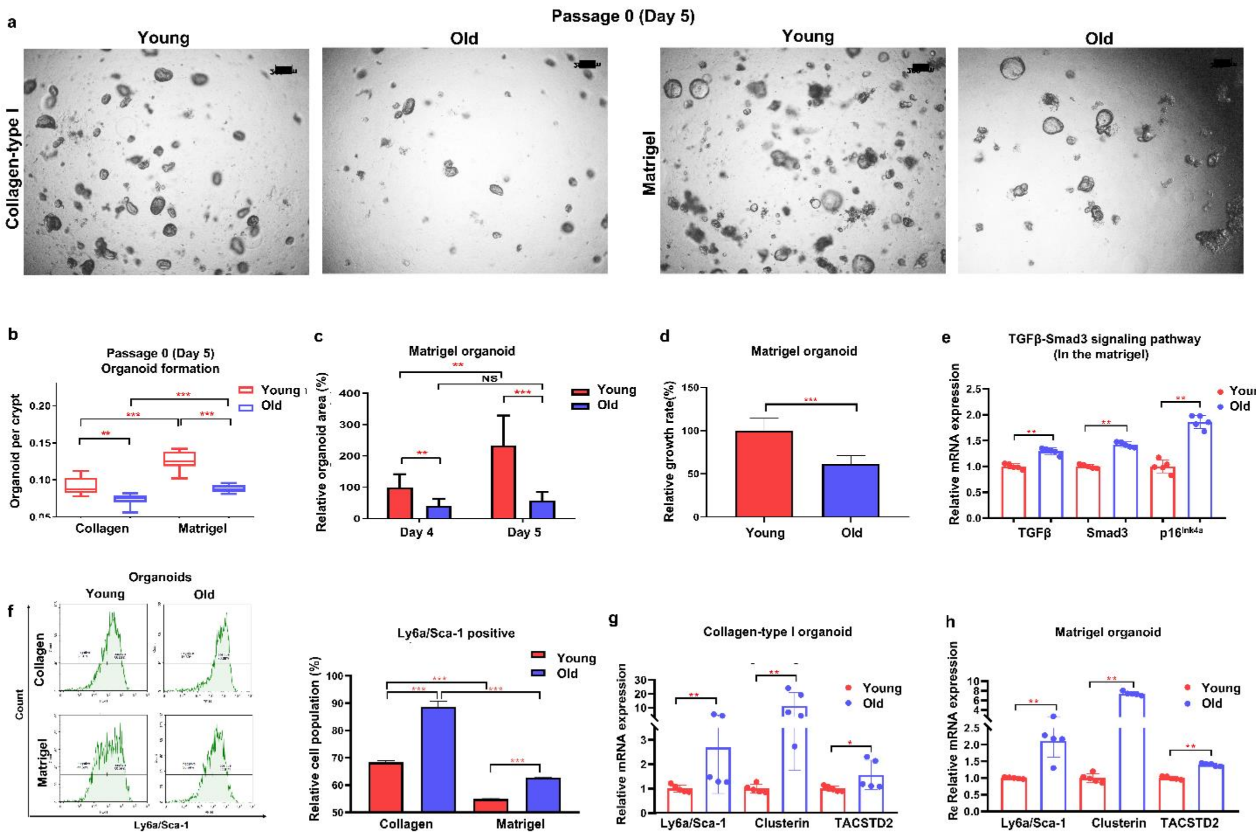

Supplementary Fig. 3 The effect of aging on the formation of colonic epithelial organoids is not dependent on the culture condition (**a**) Representative images of young- and old-mouse organoids on day 5 of passage 0 after seeding in the collagen type I and Matrigel. The scale bar represents 200 μm. (**b**) The number of organoids derived from young and old mice cultured at an average density of 500 crypts per well in each group. Data are reported as the mean ± standard deviation (SD) of 8 wells. (**c**) The relative organoid area of young and old mice at days 4 and 5 in the Matrigel condition. The represented percentage is relative to young-mouse organoids on day 4. Data are reported as the mean ± SD of 8 organoids (**d**) The growth rate of young- and old-mouse organoids in the Matrigel. Data are reported as the mean ± SD of 8 organoids (**e**) The mRNA expression of TGF-β, Smad3, $p16^{INK4a}$ genes in young and old-mouse organoids in the Matrigel. Data are reported as the mean ± SD (n = 5) (**f**) Flow cytometric analysis of cells from the colonic organoids on the collagen and Matrigel. Representative plots for Ly6a/Sca-1 positive cell population in the organoids (n = 3) (**g, h**) mRNA expression of fetal markers, Ly6a/Sca-1, Clusterin, and TACSTD2 signatures in young and old-mouse organoids in the collagen (n = 5) and Matrigel (n = 5). Data are reported as the mean ± SD. $^{*}p$ <0.05, $^{**}p$ <0.01, $^{***}p$ <0.001, $^{****}p$ <0.0001

A

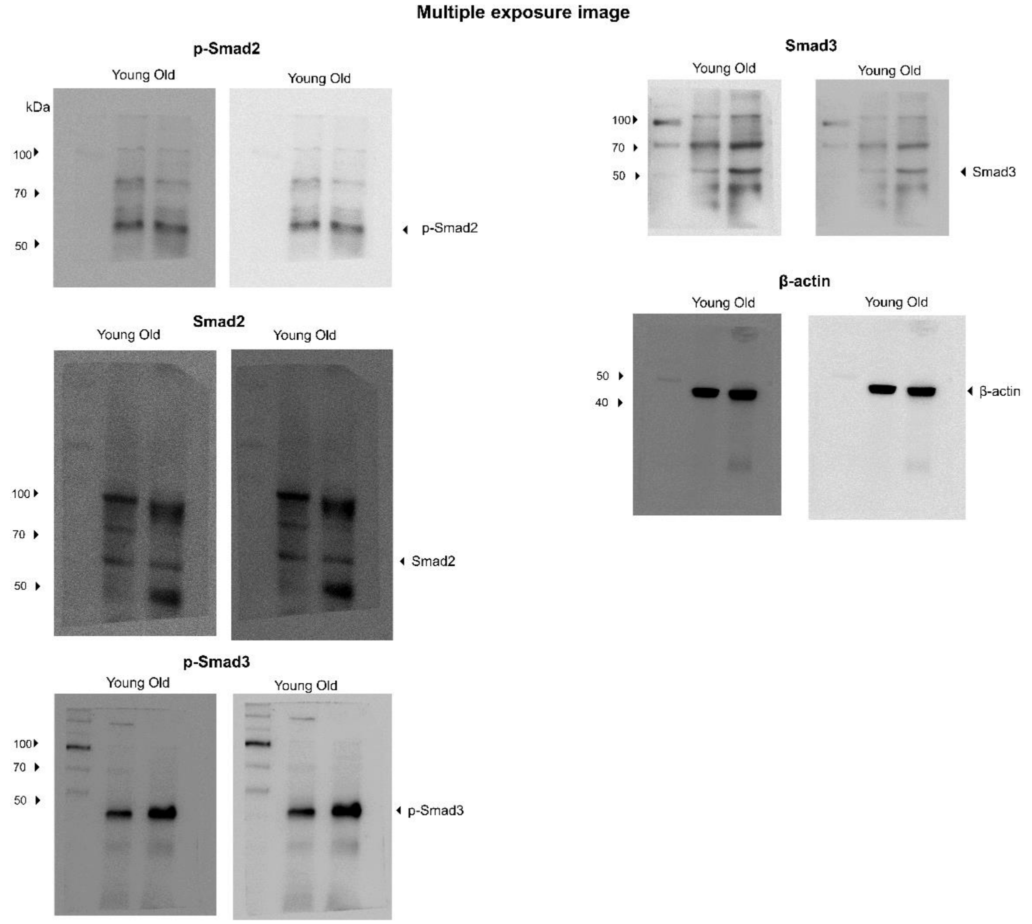

Supplementary Fig. 4 Original blots of Western blot.

(**A**) Original blots of Figure 2E. For more significant presentation purposes, we altered the aspect ratios of blots and adjusted the brightness of the blots. Multiple exposure images were provided. And every blot was cut before hybridization with antibodies.

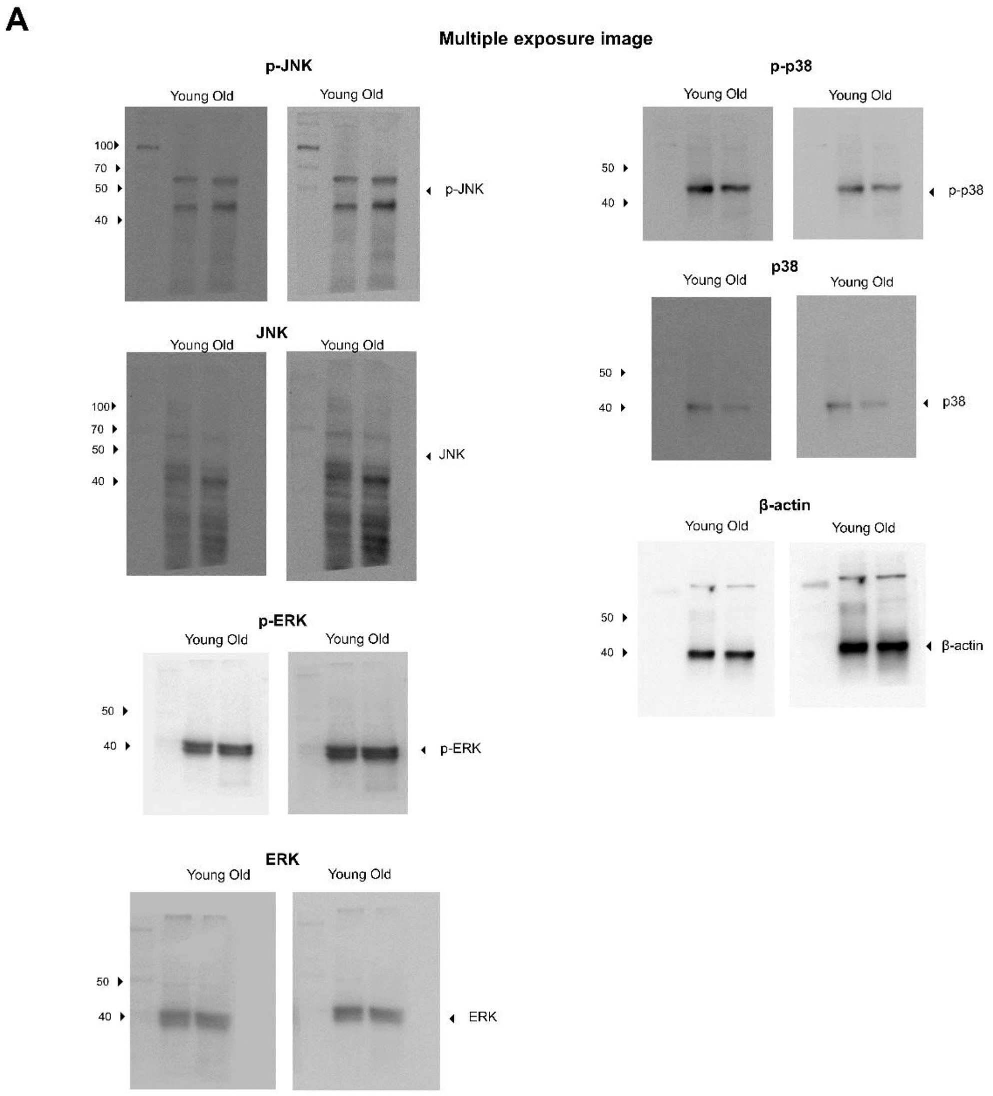

Supplementary Fig. 5 Original blots of Western blot.

(**A**) Original blots of Figure 2F. For more significant presentation purposes, we altered the aspect ratios of blots and adjusted the brightness of the blots. Multiple exposure images were provided. And every blot was cut before hybridization with antibodies.

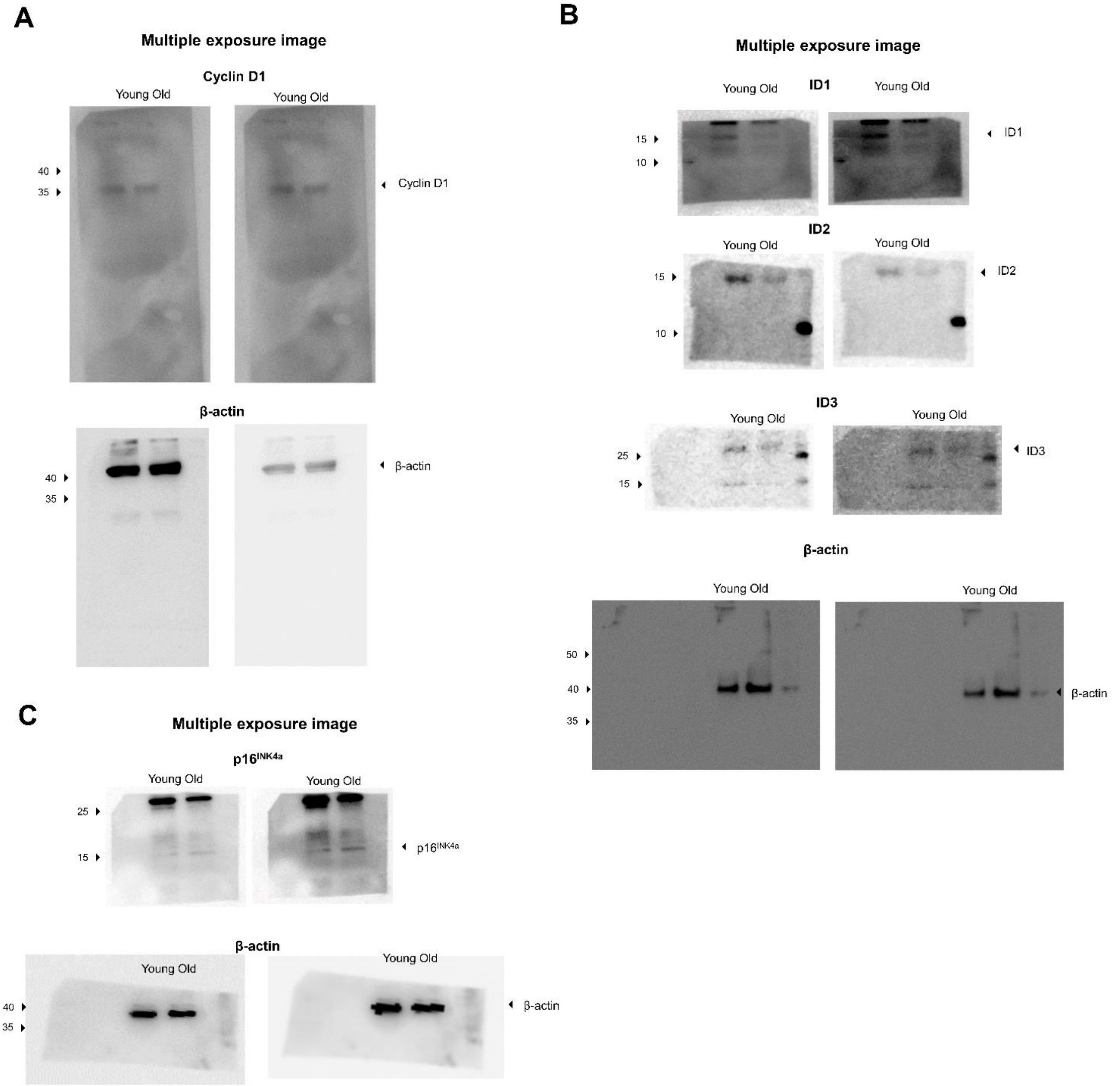

Supplementary Fig. 6 Original blots of Western blot.

(**A**) Original blots of Figure 3C. (**B**) Original blots of Figure 3F. (**C**) Original blots of Figure 3G. For more significant presentation purposes, we altered the aspect ratios of blots and adjusted the brightness of the blots. Multiple exposure images were provided. And every blot was cut before hybridization with antibodies.

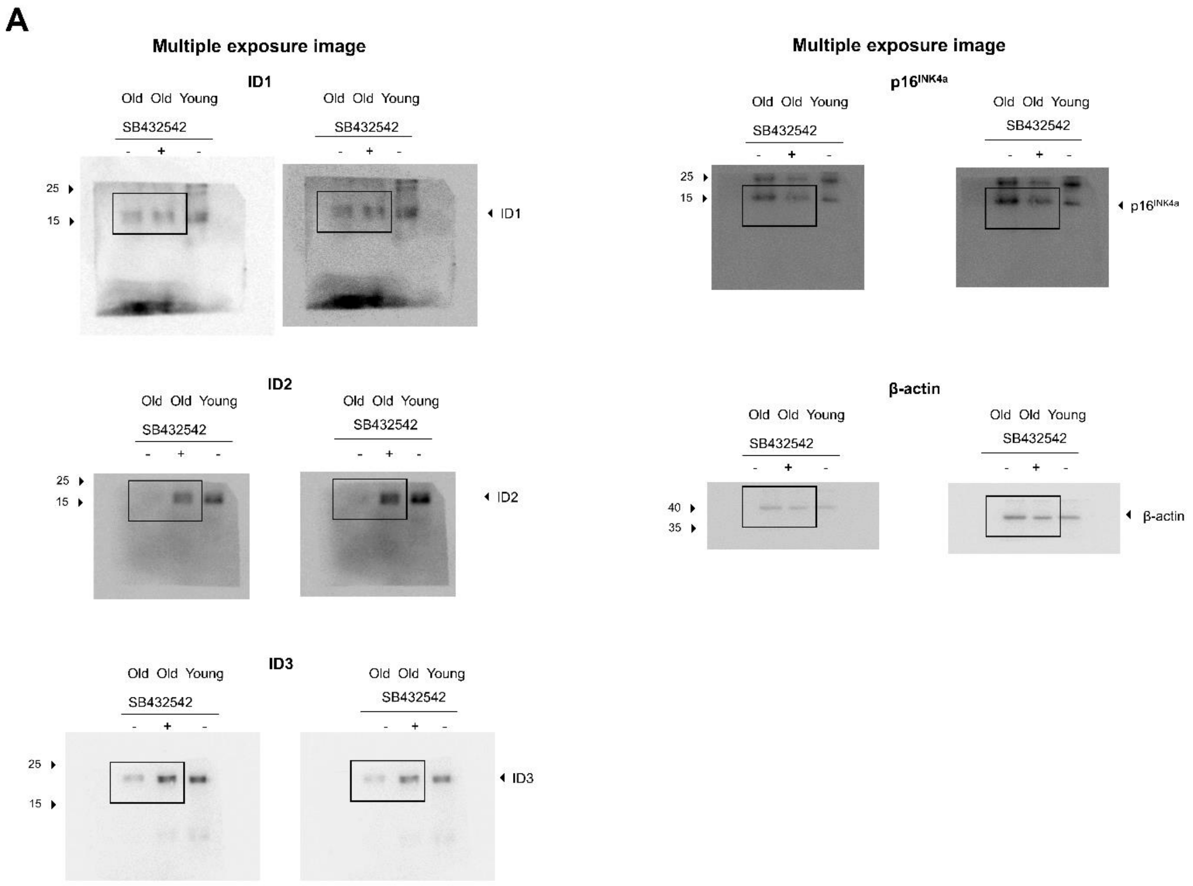

Supplementary Fig. 7 Original blots of Western blot.

(**A**) Original blots of Figure 5E. For more significant presentation purposes, we altered the aspect ratios of blots and adjusted the brightness of the blots. Multiple exposure images were provided. And every blot was cut before hybridization with antibodies.
