# Supplementary material for: Effect of aging on the formation and growth of colonic epithelial organoids by changes in cell cycle arrest through TGF-β-Smad3 signaling
Source: Inflamm Regen. 2023 Jul 13;43:35. doi: 10.1186/s41232-023-00282-6 (PMC10339613; doi:10.1186/s41232-023-00282-6)
Supplement: Supplementary file 2 — Additional file 2. [file 41232_2023_282_MOESM2_ESM.zip › 3. Supplementary Method_ESM.docx]

**Supplementary Materials and Methods for**

**“Effect of aging on the formation and growth of colonic epithelial organoids by changes in cell cycle arrest through TGF-β-Smad3 signaling”**

Min Kyoung Jo^1,2^, Chang Mo Moon^1,2*^, Hyeon-Jeong Jeon^1,2^, Yerim Han^1,2^, Eun Sook Lee^1,2^, Ji-Hee Kwon^3^, Kyung-Min Yang^4^, Young-Ho Ahn^2,5^, Seong-Eun Kim^1^, Sung-Ae Jung^1^, and Tae Il Kim^3*^

^1^Department of Internal Medicine, College of Medicine, Ewha Womans University, Seoul, Republic of Korea

^2^Inflammation-Cancer Microenvironment Research Center, College of Medicine, Ewha Womans University, Seoul, Republic of Korea

^3^Department of Internal Medicine, Yonsei University College of Medicine, Seoul, Republic of Korea

^4^Medpacto Inc., Seoul, Republic of Korea.

^5^Department of Molecular Medicine, College of Medicine, Ewha Womans University, Seoul, Republic of Korea

**Corresponding authors:**

Chang Mo Moon, MD, PhD

Department of Internal Medicine and Inflammation-Cancer Microenvironment Research Center

College of Medicine, Ewha Womans University

1071 Anyangcheon-ro, Yangcheon-gu, Seoul, 07985, Republic of Korea

Phone: +82-2-2650-2945, Fax: +82-2-2650-5936, E-mail: [mooncm27@ewha.ac.kr](mailto:mooncm27@ewha.ac.kr)

Tae Il Kim, MD, PhD

Division of Gastroenterology and Department of Internal Medicine

Yonsei University College of Medicine

50-1 Yonsei-ro, Seodaemun-gu, Seoul 03722, Republic of Korea

Phone: +82-2-2228-1965, Fax: +82-2-393-6884, E-mail: [taeilkim@yuhs.ac](mailto:taeilkim@yuhs.ac)

**Colonic organoid culture in Matrigel**

Colonic crypts were isolated from mice. A total of 500 crypts were mixed with 20 μl of collagen and plated in 48-well plates. For the RNA extraction, 50 μl of droplets of Matrigel with crypts were plated onto a flat-bottom 24-well plate. After the Matrigel polymerized, 200–500 μl of the organoid culture medium was added to each well. The medium was changed every two days. Organoids were quantified on days 4 and 5 of culture.

**Quantification of the number and growth rate of organoids**

The number of organoids (500 crypts/well) was counted at day 5 after plating on a bright-field microscope (Carl Zeiss Microimaging Inc., Germany). In the organoids from young and old mice, 5 organoids from each mouse with a spherical shape and clearly defined borders were chosen, and the areas of these organoids were measured on day 4 and 5 from images using Image J software (National Institutes of Health, Bethesda, MD). When compared the organoid size between young and old group, relative quantification was performed based on the size of young-mouse organoids on day 4. In the organoids from young and old mice, the growth rate was defined as the change in the organoid area from day 4 to day 5 (each group).

**Quantification of the number and growth rate of organoids from DSS-colitis mice**

The number of organoids (2000 crypts/well) was counted on day 7 after plating on a bright-field microscope (Carl Zeiss Microimaging Inc.). In the organoids from DSS-colitis mice, 8 organoids with a spherical shape and clearly defined borders were chosen, and the areas of these organoids were measured on day 6 and 7 from images using Image J software due to slower growth than normal organoids from young and old mice. When compared the organoid size between young and old group, relative quantification was performed based on the size of organoids from young DSS-colitis mice on day 6. In the organoids from DSS-colitis mice, the growth rate was defined as the change in the organoid area from day 6 to 7. Statistical significance was calculated using the Mann-Whitney t-test as a nonparametric test of the means for each group.

**Passage of colonic epithelial organoids**

Colonic epithelial organoids were usually cultured for one week for passage 0 and then could be passaged between four and seven days. For passaging, organoids embedded in collagen gel were incubated in a digestion medium consisting of DMEM/high glucose, penicillin/streptomycin, and 1% FBS (completed DMEM) with 6.25 mg/ml collagenase. The organoids were suspended in PBS and centrifuged and incubated in trypsin/EDTA for 5 min followed by the addition of completed DMEM. Pelleted organoids were embedded in fresh collagen and planted in wells twice as before, followed by the addition of the culture medium, which was either the normal medium or SB431542 (TGF-β inhibitor)-treated medium at a concentration of 0.5 μM.

**RNA isolation and quantitative real-time PCR**

Total RNA was extracted from mouse colon crypts, tissue, normal colonic organoids, and DSS colitis mouse colonic organoids using Trizol Reagent (Invitrogen, Grand Island, NY, USA) according to the manufacturer’s protocol. The extracted RNA was converted to cDNA with an RT reagent kit (Promega, Madison, WI, USA). Quantitative real-time PCR (qRT-PCR) was performed in a real-time PCR system using SYBR green master mix (Applied Biosystems, Foster City, CA, USA). The relative mRNA was normalized using β-actin mRNA. All qRT-PCR experiments were repeated at least five times. The mouse primers used in our experiments are shown in **Supplementary Table S1**.

**Western blot**

When colonic organoids were cultured on day 5 of passage 1, organoids were lysed in RIPA buffer (iNtRON Biotechnology, Gyeonggi, Republic of Korea) supplemented with a protein inhibitor cocktail for extracting protein. Isolated proteins were separated on 10%, 12% sodium dodecyl sulfate-polyacrylamide gel electrophoresis (SDS-PAGE), transferred to polyvinylidene fluoride (PVDF) membranes (Merk Millipore, Billerica, MA, USA), and incubated with antibodies against the Smad2, p-Ssmad2, Smad3, p-Smad3, Smad2/3, p-Smad2/3, ID1, ID2, ID3, p16^INK4a^, JNK, p-JNK, p-ERK, ERK, p-p38, p-38, Cyclin D1, Bcl-2, Bax, Caspase-3, cleaved caspase-3, and β-actin. Protein was detected using the ECL Western blotting Luminol reagent (Santa Cruz, CA, USA). Information about all the antibodies used in these experiments is shown in **Supplementary Table S2**.

**Cell cycle analysis**

Each phase of the cell cycle was stained with propidium iodide (PI; Abcam, Cambridge, MA, USA) and was evaluated using DNA flow cytometry analysis. The cell cycle analysis was performed on colon crypts treated with collagenase for 1 hr followed by trypsin/EDTA solution. The cells were fixed with 70% EtOH, and 0.2 mg/ml Rnase A and 10 μg/ml PI were added for at least 30 min; then, the cells were counted by flow cytometry at 488 nm (ACEA NovoCyte 3000 Cytometer, ACEA Biosciences Inc. San Diego, USA). The results were analyzed using a flow cytometry software program (ACEA NovoExpress, ACEA Biosciences Inc.).

**Flow cytometry**

The cells were obtained from colon crypts treated with collagenase for 1 hr, followed by trypsin/EDTA solution on the tissue. The cells were harvested from organoids treated with collagenase for 1 hr on the organoids in the collagen or treated with TrypLE for 1 hr on the organoids in the Matrigel. The cells were resuspended in flow cytometry buffer (12.5 g bovine serum albumin [BSA, Sigma-Aldrich] and 0.372 g ethylenediaminetetraacetic acid [EDTA, Sigma-Aldrich] in 500 mL PBS), and then incubated with antibodies for 45 min at 4℃. All samples were analyzed using the Novo-Cyte flow cytometer (ACEA Biosciences, San Diego, CA, USA). Information regarding the antibodies used in these experiments is shown in **Supplementary Table S2**.

**Immunohistochemistry**

Colonic epithelial organoids at day 5 of passage 0 were washed with cold PBS, fixed with 4% paraformaldehyde solution (Biosesang, Seongnam, Korea) overnight, gradually dehydrated in sucrose (Sigma-Aldrich), and embedded in optimal cutting temperature compound (OCT; Leica Biosystems, Wetzlar, Germany). The organoids were sectioned with a cryostat (Leica Biosystems) into 20–30-μm thick sections. The tissues from the colons of the mice were washed with cold PBS, fixed with 4% paraformaldehyde solution overnight, and gradually dehydrated in alcohol (Duksan Pure Chemicals, Ansan, Korea). Then, paraffin-embedded (Leica Biosystems) tissue sections were cut 5 μm thick. For immunohistochemistry (IHC), these sections were incubated with 10 mM sodium citrate buffer (pH 6.0; Sigma-Aldrich) and boiled for 8 min for antigen retrieval. Endogenous peroxidase activity was blocked by incubating the sections in 3% hydrogen peroxide (ammonia water; Duksan Pure Chemicals) diluted in PBS (Biosesang) for 30 min. These sections were then blocked with 3% bovine serum albumin (BSA; Sigma-Aldrich) in Tris-Buffered Saline (TBS; Biosesang) for 30 min. After blocking, sections were incubated with TGF-β1, Smad3, and p16^INK4a^ primary antibodies at 4℃ overnight. The antibodies used are shown in **Supplementary Table S2**. Slides were then incubated with the Vectastain ABC kit (Vector Laboratories, CA, USA) and were visualized using diaminobenzidine solution (DAB; Vector Laboratories). Slides were counterstained with hematoxylin (Sigma-Aldrich), dehydrated in a gradient of ethanol, cleared in xylene (Sigma-Aldrich), and cover-slipped with per-mounting media (Thermo Fisher Scientific). Staining was confirmed under a bright-field microscope (BX51; Olympus, Tokyo, Japan) for organoids slides and an Aperio slide scanner (Leica Microsystems, Vista, CA) for tissue slides. The slide images were evaluated using Aperio’s annotation software (ImageScope v10, Aperio). IHC scoring (from 0 to 12) is the score of intensity × the score of the percentage of stained cells. IHC scoring is shown in **Supplementary Table S3**.

**RNA sequencing library and bioinformatic analysis**

For RNA sequencing, mRNA was extracted from young- and old-mice organoids at day 7 using the Illumina TruSeq stranded mRNA Sample Preparation kit (TruSeq; Illumina, San Diego, CA, USA) according to the manufacturer’s instructions. All libraries were sequenced with a Hiseq 2500 sequencing machine (Illumina). Gene Ontology (GO), and KEGG pathway enrichment analyses were performed by using the Database for Annotation, Visualization, and Integrated Discovery (DAVID) tool (http://david.abcc.ncifcrf.gov) and the KEGG orthology-based annotation system (KOBAS) online tool (http://geneontology.org) with a cutoff value of *p* < 0.05. In addition, we generated a protein-protein interaction (PPI) network using string (www.string-db.org).

**DSS-colitis mouse experiment**

The 6-week-old male C57BL/6 mice were purchased from Raon Bio (Gyeonggi-do, Republic of Korea). The mice were given dextran sulfate sodium (DSS) (MP Biomedicals, LLC, Illkirch, France) in drinking water at a concentration of 2.5% (weight/volume) for 5 days and then drinking water for 12 days. The body weight, stool consistency, and stool blood were monitored and recorded daily to evaluate the disease activity index (DAI). Mice were sacrificed on day 16 to investigate clinical pathology and parameters. DAI scoring is shown in **Supplementary Table S4**. Colon samples were fixed with a 4% paraformaldehyde solution, embedded in paraffin, cut into 4-μm sections, stained with hematoxylin and eosin (H&E) (Abcam), dehydrated, and mounted. Images were acquired using a slide scanner (VS200, Olympus). Histological scoring is shown in **Supplementary Table S5**.

**Construction of a protein-protein interaction (PPI) network**

The interaction network among proteins encoded by candidate DEGs was researched by importing all the candidate DEGs into the STRING database (http://string-db.org) and calculating it online.
