# Supplementary material for: Effect of aging on the formation and growth of colonic epithelial organoids by changes in cell cycle arrest through TGF-β-Smad3 signaling
Source: Inflamm Regen. 2023 Jul 13;43:35. doi: 10.1186/s41232-023-00282-6 (PMC10339613; doi:10.1186/s41232-023-00282-6)
Supplement: Supplementary file 2 — Additional file 2. [file 41232_2023_282_MOESM2_ESM.zip › 5. Supplementary Tables_ESM.docx]

**Supplementary Tables for**

**“Effect of aging on the formation and growth of colonic epithelial organoids by changes in cell cycle arrest through TGF-β-Smad3 signaling”**

Min Kyoung Jo^1,2^, Chang Mo Moon^1,2*^, Hyeon-Jeong Jeon^1,2^, Yerim Han^1,2^, Eun Sook Lee^1,2^, Ji-Hee Kwon^3^, Kyung-Min Yang^4^, Young-Ho Ahn^2,5^, Seong-Eun Kim^1^, Sung-Ae Jung^1^, and Tae Il Kim^3*^

^1^Department of Internal Medicine, College of Medicine, Ewha Womans University, Seoul, Republic of Korea

^2^Inflammation-Cancer Microenvironment Research Center, College of Medicine, Ewha Womans University, Seoul, Republic of Korea

^3^Department of Internal Medicine, Yonsei University College of Medicine, Seoul, Republic of Korea

^4^Medpacto Inc., Seoul, Republic of Korea.

^5^Department of Molecular Medicine, College of Medicine, Ewha Womans University, Seoul, Republic of Korea

**Corresponding authors:**

Chang Mo Moon, MD, PhD

Department of Internal Medicine and Inflammation-Cancer Microenvironment Research Center

College of Medicine, Ewha Womans University

1071 Anyangcheon-ro, Yangcheon-gu, Seoul, 07985, Republic of Korea

Phone: +82-2-2650-2945, Fax: +82-2-2650-5936, E-mail: [mooncm27@ewha.ac.kr](mailto:mooncm27@ewha.ac.kr)

Tae Il Kim, MD, PhD

Division of Gastroenterology and Department of Internal Medicine

Yonsei University College of Medicine

50-1 Yonsei-ro, Seodaemun-gu, Seoul 03722, Republic of Korea

Phone: +82-2-2228-1965, Fax: +82-2-393-6884, E-mail: [taeilkim@yuhs.ac](mailto:taeilkim@yuhs.ac)

**Supplementary Table S1. List of primer sequences for real-time qPCR.**

| **primers** | **Forward (5’ - 3’)** | **Reverse (5’ - 3’)** |
| --- | --- | --- |
| **TGF-β1** | TGTACAGCTGCCGCACACA | TGTACAGCTGCCGCACACA |
| **Smad2** | AACCCGAATGTGCACCATAAGAA | GCGAGTCTTTGATGGGTTTACGA |
| **Smad3** | GTCAACAAGTGGTGGCGTGTG | GCAGCAAAGGCTTCTGGGATAA |
| **Smad4** | TGACGCCCTAACCATTTCCAG | CTGCTAAGAGCAAGGCAGCAAA |
| **Smad7** | AGAGGCTGTGTTGCTGTGAATC | CCATTGGGTATCTGGAGTAAGGA |
| **ID1** | ATCGCATCTTGTGTCGCTGAG | AGTCTCTGGAGGCTGAAAGGT |
| **ID2** | ATGAAAGCCTTCAGTCCGGTG | AGCAGACTCATCGGGTCGT |
| **ID3** | CAGGGTCCCAAGCGAACGG | TTGCCACTGACCCGGTCGTC |
| **p16INK4a** | TACCCCGATTCAGGTGATGATG | TAGCTCTGCTCTTGGGATTGG |
| **p15INK4b** | AGATCCCAACGCCCTGAAC | CCCATCATCATGACCTGGATT |
| **p18INK4c** | AAATGGATTTGGGAGAACTGC | AAATTGGGATTAGCACCTCTGA |
| **p19INK4d** | ACACCTGTCCATTGAAGAAGG | CCCCAAACACACACACTCAA |
| **p19ARF** | GGGTTTTCTTGGTGAAGTTCG | TTGCCCATCATCATCACCT |
| **p21Cip1** | CGAGAACGGTGGAACTTTGAC | TCCCAGACGAAGTTGCCC |
| **p27Kip1** | GTTAGCGGAGCAGTGTCCA | TCTGTTCTGTTGGCCCTTTT |
| **Lgr5** | CGGGACCTTGAAGATTTCCT | GATTCGGATCAGCCAGCTAC |
| **OLFM4** | CGAGACTATCGGATTCGCTATG | TTGTAGGCAGCCAGAGGGAG |
| **PDGFA** | GTAACACCAGCAGCGTCAAGT | TGGCTTCTTCCTGACATACTCC |
| **AQP4** | TTTCTCCCATACTGCTTTGCC | AGAAAGCCCTCCATTACCAGC |
| **MSI1** | CCAGGGTTCCAAGCCACGA | CCATAAGCCGTGAGAGGGATA |
| **Sox9** | GCCAGATGGACCCACCAGTAT | TCCAAACAGGCAGGGAGATTC |
| **CDCA7** | ATGTCATCAGTGTCGCCAGAAA | CCTCGCCATAGCGGTTTCG |
| **SMOC2** | CCCAAGCTCCCCTCAGAAG | GCCACACACCTGGACACAT |
| **BMP2** | GGGACCCGCTGTCTTCTAGT | TCAACTCAAATTCGCTGAGGAC |
| **BMP4** | TTCCTGGTAACCGAATGCTGA | CCTGAATCTCGGCGACTTTTT |
| **BMPR1A** | GGACCAGAAGAAGCCAGAAA | CTTTCGGTGAATCCTTGCAT |
| **BMPR1B** | TGACTCTGGAATGCCTGTTG | AGAGTGGGGTGGAGGTCTTT |
| **Smad1** | GGGGCCGAGCTGCTAAT | CTATGAAACAGAAGAAATGGGG |
| **Smad5** | GTGGAAACAGGGCGATGAAG | GCGTCCACAGCTTTCTCTGC |
| **Smad8** | GCAGTGCCTTGGGGTTTTCA | AAGCAGTGGGGTCCGATTCAA |
| **Ly6a/Sca-1** | TCAGGAGGCAGCAGTTATTGTGGA | TACATTGCAGAGGTCTTCCTGGCA |
| **Clusterin** | CGGGCGTCTGGCATCATA | CATGCGGCTTTTCCTGCG |
| **TACSTD2** | GAAAGGGACATTAAAGGCGAGT | ACCGAGACGACAGCGATG |
| **β-actin** | TGGAGAAGAGCTATGAGCTGCCTG | GTGCCACCAGACAGCACTGTGTTG |

**Supplementary Table S2. List of antibodies**

| **Name** | **Application** | **Species** | **Company** |
| --- | --- | --- | --- |
| **TGF-β1** | Immunohistochemistry (1:400) | Mouse | R&D Systems |
| **Smad 2** | Immunoblotting (1:1000) | Mouse | Cell signaling |
| **p-Smad 2** | Immunoblotting (1:1000) | Rabbit | Cell signaling |
| **Smad 3** | Immunoblotting (1:1000) | Rabbit | Abcam |
|  | Immunohistochemistry (1:500) |  |  |
| **p-Smad 3** | Immunoblotting (1:1000) | Rabbit | Abcam |
| **Smad-2/3** | Immunoblotting (1:1000) | Rabbit | Cell signaling |
| **p-Smad 2/3** | Immunoblotting (1:1000) | Rabbit | Cell signaling |
|  | Immunohistochemistry (1:400) |  |  |
| **ID1** | Immunoblotting (1:200) | Mouse | Santa Cruz |
| **ID2** | Immunoblotting (1:200) | Mouse | Santa Cruz |
| **ID3** | Immunoblotting (1:200) | Mouse | Santa Cruz |
| **p16INK4a** | Immunoblotting (1:1000) | Rabbit | Bioworld |
|  | Immunohistochemistry (1:400) |  |  |
| **β-actin** | Immunoblotting (1:1000) | Rabbit | GeneTex |
| **JNK** | Immunoblotting (1:1000) | Rabbit | Cell signaling |
| **p-JNK** | Immunoblotting (1:500) | Mouse | Santa Cruz |
| **ERK** | Immunoblotting (1:1000) | Rabbit | Cell signaling |
| **p-ERK** | Immunoblotting (1:1000) | Rabbit | Cell signaling |
| **p-38** | Immunoblotting (1:1000) | Rabbit | Cell signaling |
| **p-p38** | Immunoblotting (1:1000) | Rabbit | Cell signaling |
| **cyclin D1** | Immunoblotting (1:1000) | Rabbit | Cell signaling |
| **Bcl-2** | Immunoblotting (1:500) | Rabbit | Abcam |
| **Bax** | Immunoblotting (1:500) | Mouse | Santa Cruz |
| **Caspase-3** | Immunoblotting (1:1000) | Mouse | Abcam |
| **Cleaved caspase-3** | Immunoblotting (1:1000) | Rabbit | Cell signaling |
| **PE anti-mouse Ly6a/Sca-1** | Flow cytometry (1:100) | Rat | Biolegend |

**Supplementary Table S3.** **Immunohistochemical scoring**

Assignment of immunohistochemical (IHC) score according to staining intensity and percentage of positively stained area.

| **Score** | **Percentage of stained (%)** | **Intensity of staining** |
| --- | --- | --- |
| **0** | 0% no cells stained | negative |
| **1** | 1-10% of cells stained | weak |
| **2** | 11-50% of cells stained | moderate |
| **3** | 51-80% of cells stained | strong |
| **4** | 81-100% of cells stained |  |

**Supplementary Table S4.** **Disease activity index score**

Disease activity index (DAI) score used to evaluate the DSS-induced colitis. DAI index was calculated as total score (body weight loss (%) + stool consistency + occult/gross bleeding).

| **Score** | **Weight loss (%)** | **Stool consistency** | **Occult/gross bleeding** |
| --- | --- | --- | --- |
| **0** | None | Normal | Negative |
| **1** | 1 – 5% | Loose stool + | Hemoccult positive + |
| **2** | 5 – 10% | Looser stool ++ | Hemoccult positive ++ |
| **3** | 10 – 15% | Diarrhea | Hemoccult positive +++ |
| **4** | > 15% | Severe Diarrhea | Gross bleeding |

**Supplementary Table S5.** **Histologic score**

Hematoxylin and eosin (H&E) staining score was calculated as total score (crypt architecture + inflammatory cell infiltration + muscle thickening + goblet cell depletion + crypt abscess).

| **Score** | **Crypt architecture** | **Inflammatory cell infiltration** | **muscle thickening** | **goblet cell depletion** | **crypt abscess** |
| --- | --- | --- | --- | --- | --- |
| **0** | Normal | Normal | Normal | Absent | Absent |
| **1** | Slight | Slight | Slight | Present | Present |
| **2** | Moderate | Moderate | Moderate | - | - |
| **3** | Severe | Severe | Severe | - | - |

**Supplementary Table S6. Gene ontology analysis of DEGs associated with TGFβ signaling pathway.**

| **GO data** | **Gene** |
| --- | --- |
| GO:0030335~positive regulation of cell migration | RET, PTPRZ1, CCR1, PODXL, PREX1, ONECUT2, KIT, AQP1, SEMA4F, SEMA3E, ROR2, TFAP2A, PDGFC, LAMB1, TRIP6, MYLK, CSF1R |
| GO:0006955~immune response | H2-Q2, CCL2, CXCL5, CCR1, H2-Q1, TNFSF15, CCL9, CTSS, SECTM1B, SECTM1A, VAV1, CCL28, CCL7, CD74, CCL6, TNFSF10, CCL20, IRF8, CMA1, TGTP1 |
| GO:0060326~cell chemotaxis | NOV, CCL2, CXCL5, CCL20, SAA1, CCL9, KIT, CCL28, CCL6 |
| GO:0007568~aging | CDKN1C, LRP1, CCL2, CDKN2A, KRT16, FADS1, TGFB3, MMP7, ABAT, ENO3, PPARGC1A, PCK1 |
| GO:0050729~positive regulation of inflammatory response | ACE, CCL2, PDE2A, CCL9, CTSS, CCL7, CCL6 |
| GO:0032091~negative regulation of protein binding | IFIT2, ACE, CDKN2A, CKMT1, NFATC4, LRRK2 |
| GO:0009887~organ morphogenesis | FGFR2, VDR, PAX9, HOXA13, GATA5, THRB, TGFB3, PDGFC |
| GO:0048565~digestive tract development | FGFR2, GATA5, TGFB3, PDGFC, KIT |
| GO:0042127~regulation of cell proliferation | FGFR2, SGK1, SGK3, CXCL5, FA2H, PTGS1, PLA2G2A, MMP7, NR3C2, JAG2, NOS2, CELA1 |
| GO:0002009~morphogenesis of an epithelium | KRT6A, FGFR3, KRT16, CITED1 |
| GO:0030855~epithelial cell differentiation | FGFR2, MUC1, SULT1B1, CES1D, KRT4, NEUROG3 |
| GO:0035019~somatic stem cell population maintenance | FGFR3, CDKN2A, PLA2G2A, KIT, TCF7L |
| GO:0030277~maintenance of gastrointestinal epithelium | RBP4, MUC2, MUC13 |
| GO:0050680~negative regulation of epithelial cell proliferation | FGFR2, CDKN1C, FGFR3, IFT57, PLA2G2A, KRT4 |
| GO:0012501~programmed cell death | P2RX7, GSDMC4, GSDMC2, GSDMC3 |

According to GO functional annotation, a set of DEGs were significantly enriched in some molecular functions including aging (eg, CDKN1C, CDKN2A, TGFB3), regulation of cell proliferation (eg, CDKN1C), organ morphogenesis (eg, TGFB3), digestive tract development (eg, TGFB3), maintenance of gastrointestinal epithelium (eg, MUC2, MUC13), programmed cell death (eg, P2RX7), and negative regulation of protein binding (eg, CDKN2A). DEGs were associated with TGFβ and cell cycle related genes.
